# Proteomic organ-specific signatures, ageing traits, disease risks and genetic architecture in an East Asian population

**DOI:** 10.1016/j.ebiom.2026.106401

**Published:** 2026-07-29

**Authors:** Derrick A. Bennett, Baihan Wang, Sihao Xiao, Neil Wright, Ahmed Edris, Yunhe Wang, Iona Y. Millwood, Robin G. Walters, Huaidong Du, Ling Yang, Yiping Chen, Daniel Avery, Dan Valle Schmidt, Canqing Yu, Dianjianyi Sun, Jun Lv, Michael Hill, Liming Li, Robert Clarke, Zhengming Chen, China Kadoorie Biobank Collaborative Group, Junshi Chen, Zhengming Chen, Rory Collins, Liming Li, Jun Lv, Richard Peto, Robin Walters, Canqing Yu, Dianjianyi Sun, Derrick Bennett, Daniel Avery, Maxim Barnard, Derrick Bennett, Ruth Boxall, Ka Hung Chan, Yiping Chen, Zhengming Chen, Jonathan Clarke, Huaidong Du, Ahmed Edris Mohamed, Hannah Fry, Yani Huang, Pek Kei Im, Andri Iona, Christiana Kartsonaki, Kshitij Kolhe, Hubert Lam, Kuang Lin, Iona Millwood, Sam Morris, Qunhua Nie, Sara Perez Vizan, Thu Pam, Alfred Pozarickij, Maryam Rahmati, Paul Ryder, Maruf Sarder, Dan Valle Schmidt, Roshi Shrestha, Becky Stevens, Robin Walters, Lin Wang, Neil Wright, Ling Yang, Xiaoming Yang, Pang Yao, Liming Li, Jun Lv, Canqing Yu, Dianjianyi Sun, Yuanjie Pan, Yuting Han, Can Hou, Qingmei Xia, Chao Liu, Pei Pei, Lang Pan, Xiao Han, Honglu Bian, Xinxin Chen, Zengchang Pang, Ruqin Gao, Kunzheng Lv, Shanpeng Li, Haiping Duan, Shaojie Wang, Yongmei Liu, Ranran Du, Liang Cheng, Xiaocao Tian, Hua Zhang, Dan Hu, Xiaoyan Zheng, Yujie Wang, Wei Sun, Shichun Yan, Yong Zhou, Chi Wang, Zhenyuan Wu, Lishun Zhai, Zhaoxi Pang, Shiwen Dong, Li Liu, Dapeng Yin, Bin He, Ying Liu, Xingren Wang, Tingting Ou, Xiangyang Zheng, Dewei Zheng, Shuai Yang, Lihui Li, Xingjiao Chen, Yan Xu, Jinyi Zhou, Ran Tao, Jian Su, Xikang Fan, Xuejia Chen, Yuxiao Huang, Yan Lu, Yujie Hua, Li Xing, Shuxian Wang, Jianrong Jin, Juping Ma, Kaifei Zhu, Hongfu Ren, Xingfeng Shen, Ge Zhong, Wei Mao, Zhenzhen Lu, Yanxu Zhong, Lifang Zhou, Rong Pan, Jian Lan, Xiaoping Tan, Jinxue Tan, Yishan Xie, Liuping Wei, Liyuan Zhou, Sisi Wang, Xianping Wu, Ningmei Zhang, Xiaofang Chen, Xiaoyu Chang, Zhuo Wang, Yujin He, Mingqiang Yuan, Ling Wang, Xiaofang Chen, Zhaodong Wang, Qiang Sun, Yang Lin, Faqing Chen, Xiaolan Ren, Lijun Chang, Feiming Zhong, Jianjun Feng, Weijie Hu, Xiaofang Zhang, Yalin Chen, Honghong Wang, Jun Wang, Linqi Diao, Zhiwei Han, Dengjun Zhu, Kai Kang, Shixian Feng, Wenjie Yang, Huizi Tian, Yali Yan, Bing Han, Li Gao, Shaofang Li, Tianfang Xing, Wei Tang, Lei Fan, Xiaolin Li, Huarong Sun, Xiaocong Zhao, Ying Li, Chen Hu, Pan He, Xukui Zhang, Yuanyuan Jin, Lan Luo, Min Yu, Ruying Hu, Hao Wang, Weiwei Gong, Jieming Zhong, Meng Wang, Chunxiao Xu, Keqing Gong, Hao Xu, Yuan Cao, Kaixu Xie, Lingli Chen, Xiaomei Tu, Junlong Pan, Xiaojun Li, Li Yin, Huilin Liu, Yuan Liu, Lei Yin, Xian Xie, Jing Wang, Bo Xiao, Zongwei Deng, Yuan Peng, Libo Zhang, Chan Qu, Li Deng, Qili Jiang, Yanling Chen

**Affiliations:** a Clinical Trial Service Unit (CTSU), Nuffield Department of Population Health, University of Oxford, Oxford, UK; b Broad Institute of MIT and Harvard, Cambridge, MA, USA; c National Institute on Drug Dependence and Beijing Key Laboratory of Drug Dependence, Peking University, Beijing, China; d Department of Epidemiology & Biostatistics, School of Public Health, Peking University, Beijing, China; e Peking University Center for Public Health and Epidemic Preparedness and Response, Beijing, China; f Key Laboratory of Epidemiology of Major Diseases (Peking University), Ministry of Education, Beijing, China

**Keywords:** Biological ageing, Organ-specific, Proteomics, Disease, Traits, East Asian

## Abstract

**Background:**

Protein-based ageing clocks can be used to help identify individuals at high risk of death or morbidity. There is a lack of evidence in non-European populations on clocks derived from combining different proteomic platforms and their relationship with age-related traits, diseases and genetic architecture.

**Methods:**

The prospective China Kadoorie Biobank assayed proteins via Olink and SomaScan in ∼4000 individuals with a mean age of 58 years. We used organ-enriched plasma proteins identified from the Genotype-Tissue Expression Project and trained Light Gradient Boosting models on chronological age (ChronAge) to derive protein organ age and age gaps (ProtAgeGap) for 18 organs. We then investigated their relationship with age-related traits and incident diseases after adjustment for multiple testing. Moreover, we conducted genome-wide association studies to identify genetic variants for overall and organ-specific ProtAgeGaps.

**Findings:**

The overall proteomic and organ-specific ageing clocks for each platform combined for all participants were strongly related with ChronAge. The organ-specific and overall proteomic age were associated with age-related traits and a range of incident diseases independent of ChronAge. In particular, the kidney organ-specific ageing clock was associated with higher risk of stroke (1.03; 1.02–1.05), chronic liver disease (CLD, 1.11; 1.05–1.18); chronic kidney disease (CKD, 1.19; 1.11–1.27) per 1-year higher ProtAgeGap. GWAS of overall proteomic age identified *AFF3* and *IL1RAPL1* as associated with biological ageing.

**Interpretation:**

Advanced proteomic ageing, in particular kidney organ ageing, was associated with age-related traits and diseases and, pending further validation, may have utility as a potential biomarker for clinical trials.

**Funding:**

British Heart Foundation.

**Research in context**

**Evidence before this study**

Our literature search in PubMed, using the keywords ((proteomic OR proteome) AND (ageing OR ageing OR age) AND (clock OR signature OR age predictor)) for articles published in English up to July 2026 found that initial studies investigating the associations of proteomic age-clocks with age-related traits and diseases were mostly conducted in populations of European ancestries. Later studies identified that subsets of plasma proteins are enriched for specific organ origins. More recent work has used these organ-enriched protein sets, combined with machine-learning approaches, to estimate organ-specific biological ages from blood proteomes using a single specific proteomic platform. Collectively, existing human evidence, in mostly European ancestry populations, suggests that organ-specific ageing clocks are feasible, reproducible across cohorts, and may be clinically informative at a population level.

**Added value of this study**

We demonstrate that an overall and 18 organ-specific ageing clocks (including systems such as the brain, heart, liver, and kidney), derived using a combination of two-affinity based proteomic platforms, are associated with age-related traits and incident diseases in a non-European population. We were able to show that organ-specific ageing measures add predictive value beyond chronological age and traditional clinical risk factors for both organ-specific disease incidence and all-cause mortality in our study. Moreover, we were able to perform genome-wide association studies of overall and organ-specific proteomic age gaps and identify potential new pathways for future investigation.

**Implications of all the available evidence**

At the organ-specific level, accelerated kidney ageing was linked to multiple age-related traits and diseases, not just chronic kidney disease. Generally, organ-specific ageing clocks may have potential to contribute to personalised risk assessment, mechanistic investigations and as surrogate endpoints in clinical trials.

## Introduction

Worldwide it is projected that one in six people will be aged 65 years or older by 2050, almost twice as high as in 2019.^1^ As human lifespans extend and global populations age, age-related mortality and disabilities are rising because older age is a major risk factor for various common diseases,^2^ and these ageing-related diseases accounted for more than half of the global disease burden in 2017.^3^ In China, the ageing population poses a substantial challenge, where over 360 million people are expected to be over the age of 65 by 2050, constituting 26% of its total population.^1^

Compared to chronological age (ChronAge), biological age can more accurately measure an individual’s functional capability.^4^ As proteins are the functional units of the cell, proteomic ageing clocks may provide insights into the downstream effects of genetic regulation, environmental influences, and functional outcomes of ageing.^5^^,^^6^ Lehallier et al. trained and validated an ageing clock based on SomaScan proteins in 4 K participants, and reported a high correlation (0.9) between proteomics-predicted age (ProtAge) and ChronAge across internal and external validation datasets.^7^ Likewise, Argentieri et al. validated a proteomic ageing clock using Olink proteins and demonstrated associations with mortality, age-related diseases and multi-morbidity.^8^ However, these conventional overall proteomic ageing models are agnostic to the variability of ageing across organs,^9^ and it is known that different organs are likely to age at different rates. Evidence has emerged from primarily European ancestry populations that proteomics can be used to capture the ageing of individual organs reliably and that accelerated organ-specific ageing can be linked to specific age-related diseases.^10^, ^11^, ^12^

Differences in socio-cultural factors, lifestyles, and genetics, mean that the relationships between plasma proteins and ageing are likely to differ between populations.^13^ Currently, there is a lack of research on organ-specific and overall proteomic ageing clocks derived in the Chinese population and their relationship with specific age-related traits and diseases. Moreover, while proteins from different proteomic platforms have been employed to build ageing clocks separately, no studies have combined proteins from different platforms, which may offer novel and relevant complementary information on biological ageing and improve predictive performance.^14^

This report aims to utilise data from a Chinese study to: (i) develop overall and organ-specific ageing clocks based on ∼9500 plasma proteins measured using both Olink and SomaScan platforms; (ii) compare the performance of these ageing clocks between different platforms and in combination; (iii) assess the relationship of the ageing clocks with age-related traits and diseases; and (iv) uncover the genetic architecture of a combined Olink and SomaScan overall and organ-specific proteomic ageing clocks.

## Methods

### Study population

The China Kadoorie Biobank (CKB) is a prospective cohort study of >512,000 adults recruited during 2004–2008 from 10 geographically diverse areas in China.^15^^,^^16^ At baseline, information including socio-demographics, medical history, lifestyles, and physiological measures were collected. Sex data were self-reported by study participants. Non-fasting blood samples were collected, processed, aliquoted, and then stored in liquid nitrogen. The long-term health of the participants was monitored via linkage with death and disease registries, as well as national health insurance systems recording hospitalisation episodes.^15^^,^^16^ A 5% random sample of survivors in CKB also attended a 2nd resurvey in 2013–2014 (∼8 years after baseline),^17^ and underwent a repeat interview, as well as additional assessments.

The present analysis involved a case-cohort study^18^ on proteomics of 3977 unrelated participants (1951 incident ischaemic heart disease (IHD) cases and 2026 subcohort participants), who were genotyped and had no prior history of cardiovascular diseases,^19^ as shown in Supplementary Fig. S1. Characteristics of study participants at baseline and resurvey by case-subcohort status are reported in Supplementary Table S1.

### Proteomic assays

10 ml blood samples were collected in EDTA tubes and transported in cool boxes to the regional laboratories for centrifugation (2100 g at 4 °C for 15 min) and separation into plasma and buffy coat aliquots, before being frozen for long term storage. The median (IQR) time between blood collection and centrifugation was 10.1 (13.8) hours. Stored plasma samples of the 3977 participants at baseline were retrieved from liquid nitrogen and thawed, and protein levels were measured with both the Olink Explore 3072 and the SomaScan v4.1 assays. For Olink, 40 μl plasma was aliquoted into 96-well plates (including 8 wells per plate for external QC samples). Plates were shipped for assaying at Olink Laboratories in Uppsala, Sweden (batch 1) and Boston, USA (batch 2). Protein levels were normalised based on inter-plate controls and transformed using a pre-determined correction factor. Protein levels were provided in the arbitrary Normalised Protein eXpression (NPX) unit on a log2 scale. Among a total of 2941 protein reagents, six were repeated across all four panels, resulting in 2923 unique reagents. Only one measure for each duplicated Olink reagent was used for the comparative analysis, as replicate protein levels had high correlations between panels (r > 0.8).^20^

For SomaScan, 60 μl plasma aliquots in 2D-barcoded microtubes were sent for assaying at the Somalogic Laboratory in Colorado, USA. A total of 7596 aptamers (i.e., SOMAmers) were included in the assay, with 7289 targeting human proteins. Samples were randomised at the Somalogic laboratory and aliquoted into 96-well plates (11 wells allocated for external control samples, including 5 calibrator, 3 QC, and 3 buffer samples). For 91 participants with higher sample volumes, samples were split and processed in duplicate. Only one measure from each duplicated sample was used here, as protein levels between duplicate samples were highly correlated (median rho > 0.8). The raw SomaScan assay results were standardised based on external control samples to control for variability in microarrays and variation within and across plates. The current study used the SomaScan measurements after adaptive normalisation by maximum likelihood to an external reference, which controls for inter-sample variability.^21^ The final protein levels were supplied in relative fluorescence units (RFU), which were further log2 transformed for subsequent analyses. One subcohort participant was excluded due to insufficient sample volume for SomaScan, resulting in a total of 3976 participants for final analysis.

### Genotyping and imputation

All participants included in the current study were genotyped as part of a larger genomics study in CKB,^22^ involving 100,706 participants. They were genotyped using a custom Affymetrix array, with 531,565 variants passing quality control (QC). They were converted to genome build 38 using CrossMap v0.6.1,^23^ and checked for consistency by reversing the process (liftUnder). Variants were excluded if they were not mapped, mapped to different chromosomes, or not mapped back to the same locations after liftUnder, leaving 531,542 remaining variants for further analyses. They were pre-phased using SHAPEIT v4.2 (SHAPEIT v2.904 for chromosome X)^24^ and uploaded to the TOPMed^25^ or Westlake Biobank for Chinese^26^ server for imputation. Two sets of the imputed data were merged by selecting the imputed genotype with a higher imputation INFO score for each variant. More details are given in the Supplementary Methods.

### Statistics

#### Development of proteomic ageing clocks

We followed the methodology of Oh et al. to identify organ-enriched proteins based on the Genotype-Tissue Expression Project (GTEx) RNA-seq database.^10^^,^^27^ In brief, gene expression data per tissue were first normalised using the DESeq2 R package.^28^ A protein was deemed to be enriched if its gene expression in one organ was four times higher than in other organs.^29^ We trained LightGBM^30^ models based on each participant’s plasma protein levels to predict their ChronAge, defined by their age at recruitment. We considered all available proteins in the overall model, while only the organ-enriched proteins were included in the organ-specific models. After randomly dividing 3976 participants into 70%/30% train/test splits, we first performed hyperparameter tuning using five-fold cross-validation within the 70% training data. Second, using the hyperparameters that led to the best performance in the previous step, we trained a LightGBM model using all of the 70% training data. Third, using the trained LightGBM model, we predicted the ChronAge of each participant based on their ProtAge in the 30% holdout test dataset. Finally, to assess the model’s performance, we calculated R^2^ (i.e., variance of ChronAge explained by ProtAge) in the 30% holdout test data.^8^ After the final model assessment, we performed a further five-fold cross-validation with the tuned hyperparameters to generate ProtAge for all participants. We then quantified the deviation of participants’ ProtAge from ChronAge (i.e., ProtAgeGap) by subtracting their ChronAge from ProtAge.

**Fig. 1:**
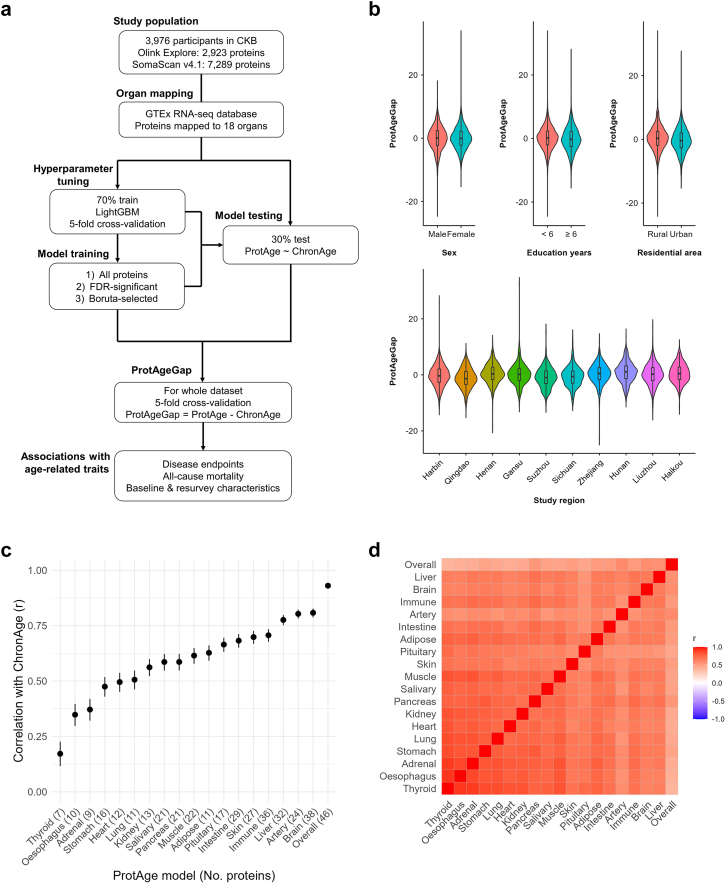
**Study design and characteristics of proteomic age.** a. Study design and analytical approach. b. Distribution of overall ProtAgeGap by socio-demographic group in all participants (n = 3976). ProtAgeGap was adjusted for chronological age, age^2^, sex, and study region, unless the variable was plotted as the x-axis. c. Correlations between overall/organ-specific ProtAge and ChronAge in holdout test data (n = 1193). d. Pairwise correlations between overall/organ-specific ProtAgeGaps in holdout test data (n = 1193). All ageing clocks shown here were trained based on both Olink and SomaScan proteins after Boruta feature selection. CKB: China Kadoorie Biobank. LightGBM: Light Gradient-Boosting Machine. ProtAge: proteomic age predicted by LightGBM. GTEx: Genotype-Tissue Expression (GTEx) Project. FDR: false discovery rate. ProtAge: Proteomic Age. ChronAge: Chronological age at recruitment. ProtAgeGap: Difference between ProtAge and ChronAge.

The overall and organ-specific models were trained based on using three subsets of data: (i) all eligible proteins; (ii) proteins significantly associated with ChronAge, after correction for multiple testing with a false discovery rate (FDR) = 0.05 (identified within the 70% training data); (iii) proteins predictive of ChronAge, selected by Boruta feature selection (identified within 70% training data; main model). The analyses were based on: i) proteins measured by both Olink and SomaScan; ii) proteins measured by Olink only; iii) proteins measured by SomaScan only. The performance of each model and pairwise correlations were evaluated in the 30% holdout test data (Fig. 1a). We also performed an external replication analysis using the CKB-trained overall and organ-specific models to assess their performance in ∼50,000 participants in the UK Biobank using Olink proteins.

#### Associations of ProtAgeGap with age-related diseases

The outcomes (defined based on ICD-10 codes) included IHD (I20–I25), stroke (I60–I61, I63–I64), diabetes (E10-E14), chronic liver disease (CLD: B18, B19, B25.1, B58.1, B94.2, K72–K77, Z22.5), chronic kidney disease (CKD: N02–N03, N07, N11, N18), and all-cause mortality. For the analysis of IHD, we excluded 31 cases that were selected but had missing information on the index event date.

Prentice-weighted Cox regression was used to estimate the adjusted hazard ratios (HRs) and 95% CIs for IHD, accounting for the bias introduced by the case-cohort design,^31^ with conventional Cox regression models used for the other outcomes. All models were adjusted for age, age^2^, sex, region, education level, smoking, alcohol drinking, physical activity, body mass index, prevalent diabetes, and hypertension at baseline. Participants with prevalent diabetes, CLD and CKD at baseline were excluded from their corresponding analyses respectively.

#### Associations of ProtAgeGap with age-related traits

Age-related traits were grouped into three categories: (i) physiological measurements at baseline (e.g., BMI); (ii) age-related traits at baseline (e.g., frailty index); and (iii) age-related traits at 2nd resurvey (e.g., broadband ultrasound attenuation [BUA], carotid intima-media thickness [CIMT], and left ventricular hypertrophy [LVH]). A frailty index based on the accumulation of deficits approach was computed by considering medical condition symptoms, signs, and physical measurements at baseline, as described in a previous publication.^32^ BUA and stiffness index were measured based on a quantitative ultrasound method for bone mineral density.^33^ Hand grip strength was defined as the average grip strength of both the left and right hands. CIMT and carotid plaque score were measured based on carotid ultrasound examinations.^17^ Bradycardia/heart block, ischaemia, and LVH were defined based on electrocardiography.^34^

Associations of overall and organ-specific ProtAgeGaps with age-related traits were investigated using linear (for continuous traits) and logistic (for binary traits) regression models, adjusted for age, age^2^, sex, region, education level, smoking, alcohol drinking, physical activity, BMI (except for analyses of adiposity-related traits), prevalent diabetes (except for analysis of random glucose), and hypertension diagnosis (except for analysis of blood pressure) at baseline.

#### Associations of ProtAgeGap with modifiable risk factors

We compared ProtAgeGaps by subgroups of lifestyle and medical history measures at baseline including smoking, alcohol drinking, and physical activity. The food diversity index was derived by summing up the intake frequency (Never = 0, 1–3 days per week = 1, 4–7 days per week = 2) across 12 different food categories. The lifestyle index was based on lifestyles and body shape with a higher score indicating a healthier lifestyle.^35^, ^36^, ^37^ Depressive and anxiety symptoms were identified using the Composite International Diagnostic Inventory-short form (CIDI-SF).^38^ We converted continuous measures into categories (based on tertiles) and sleep duration to long (>10 h), normal (6–10 h), and short sleep (<6 h) and adjusted ProtAgeGaps for age, age^2^, sex, region, education level, smoking, alcohol drinking, physical activity, BMI, prevalent diabetes, and hypertension diagnosis at baseline, where appropriate. Differences in the adjusted ProtAgeGap between subgroups of each lifestyle/medical history were assessed using ANOVA. All non-genetic analyses were conducted in R v4.4.2.^39^ and Python v3.11.3.^40^

#### Multiple testing

The main analyses used the ageing clocks trained on both proteomic platforms in the all participants included 456 tests and this was based on 19 ProtAgeGaps (1 overall + 18 organ-specific) ∗ (6 age-related diseases and all-cause mortality at follow-up + 10 age-related traits at baseline + 8 age-related traits at resurvey). The p-values from these 456 tests were adjusted for multiple testing using a false discovery rate = 0.05. We then repeated the same analyses: 1) in subcohort participants; 2) using ageing clocks trained on Olink proteins only; and 3) using ageing clocks trained on SomaScan proteins only. We applied the same approach for multiple testing correction to each of these additional analyses.

#### Discovery of genetic architecture of proteomic ageing

Finally, we performed genome-wide association studies (GWAS) on overall and organ-specific ProtAgeGaps using BOLT-LMM v2.5, which uses linear mixed models to account for population stratification and relatedness.^41^ GWAS were adjusted for age, age^2^, sex, study region, and the first 11 genomic principal components, and variants with minor allele count (MAC) < 20 (MAC = minor allele frequency ∗ INFO ∗ sample size ∗ 2) were removed. Lead variants that achieved genome-wide significance (p = 5 × 10^−8^) were identified based on a sliding window of 500 kb. SNP-based heritability was estimated using LD score regression,^42^ based on linkage disequilibrium references of the East Asian ancestry population from the 1000 Genomes Project.^43^ Post-GWAS harmonisation of summary statistics and generation of Manhattan and Q–Q plots were conducted in GWASLab.^44^

#### Sensitivity analyses

In order to assess the robustness of our model training we repeated our analyses using the models trained in the subcohort participants only in order to predict ProtAge in all participants. We then investigated the associations of these newly derived ProtAgeGaps with diseases and traits in all participants in order to assess whether the results were consistent with the main findings.

We defined accelerated agers as those participants with overall and organ-specific ProtAgeGaps > 1.5 Standard Deviations (SD) above the mean overall or organ-specific ProtAgeGap after adjustment for chronological age. Super agers were defined as participants >1.5 SD below the mean overall or organ-specific ProtAgeGap after adjustment for chronological age. We repeated the analyses to assess the associations of these two ageotypes with diseases and traits.

There is a known systematic bias in ageing clocks when the age gap is defined as the difference between the estimated biological age and chronological age.^45^ We tried to mitigate against this bias by conducting analyses whereby we regressed each of our overall and organ-specific ProtAge estimates on chronological age and defined the ProtAgeGaps using the residuals from each of these respective regressions. This approach mitigates the systematic dependence on chronological age and is the key approach used for other types of ageing clock analyses (e.g., epigenetic).^46^^,^^47^

#### Risk prediction

We conducted risk prediction of all-cause mortality and IHD events in all participants and the subcohort only order to assess the predictive ability of the overall and organ-specific ProtAgeGaps over and above conventional risk factors (age, sex, smoking, diabetes, SBP and waist circumference). Accurate predictions discriminate between those with and those without the outcome and discrimination was measured by the Harrell’s C-statistic^48^ and we used continuous, category-free Net Reclassification Index (NRI)^49^ to assess how many individuals were reclassified by individually adding the overall or organ-specific ProtAgeGaps to the model with conventional risk factors. The calibration, reflecting the agreement between observed and predicted risk, was assessed via the calibration-in-the-large approach.^50^

### Ethics

Ethical approval was obtained from the Ethical Review Committee of the Chinese Centre for Disease Control and Prevention (Beijing, China, 005/2004) and the Oxford Tropical Research Ethics Committee, University of Oxford (UK, 025-04). All participants provided written informed consent and the study was conducted in accordance with the Declaration of Helsinki principles.

### Role of funders

The funding bodies had no involvement in study design, data collection, statistical analysis, data interpretation, or manuscript preparation.

## Results

For the overall proteomic ageing clock 2168 proteins were common to both the Olink and SomaScan platforms (Supplementary Fig. S2a). There was also substantial overlap between the FDR-significant proteins and Boruta-selected proteins from both platforms respectively (Supplementary Fig. S2b and c). Compared with previous published overall proteomic ageing clock studies, we identified some unique proteins that had not been identified previously (Supplementary Fig. S3). The performance of the overall proteomic ageing clock for each platform separately and combined for all participants and the sub-cohort were broadly similar with R^2^ of ∼0.9 for ProtAge with ChronAge (Supplementary Figs. S4 and S5).

Our main analyses focused on ageing clocks trained based on both Olink and SomaScan proteins after Boruta feature selection, as these models achieved good performance in predicting ChronAge with a relatively small number of proteins. There were minor differences in the ProtAgeGaps by sex, education and urban and rural residence, as well as by the 10 CKB study regions for the two platforms combined (Fig. 1b). Patterns by ProtAgeGaps were broadly similar when the platforms were investigated separately for all participants (Supplementary Fig. S6) and in the subcohort (Supplementary Fig. S7).

There was a wide range of correlations (0.17–0.81) of the 18 organ-specific ProtAgeGaps with ChronAge, with the overall ProtAge having the strongest correlation with ChronAge (0.93) (Fig. 1c) in the holdout test dataset for both platforms combined. All of the organ-specific ProtAgeGaps were positively correlated with each other with correlations generally exceeding 0.5 (Fig. 1d). The results for the organ-specific ageing clocks were consistent when the platforms were investigated individually in all participants (Supplementary Fig. S8a–f) and in the subcohort participants only (Supplementary Fig. S9a–f). We applied the Olink organ-specific and overall proteomic ageing clocks developed in CKB to the UK Biobank Olink data and observed broadly similar correlations with ChronAge and between organ-specific ageing clocks (Supplementary Fig. S10).

**Fig. 2:**
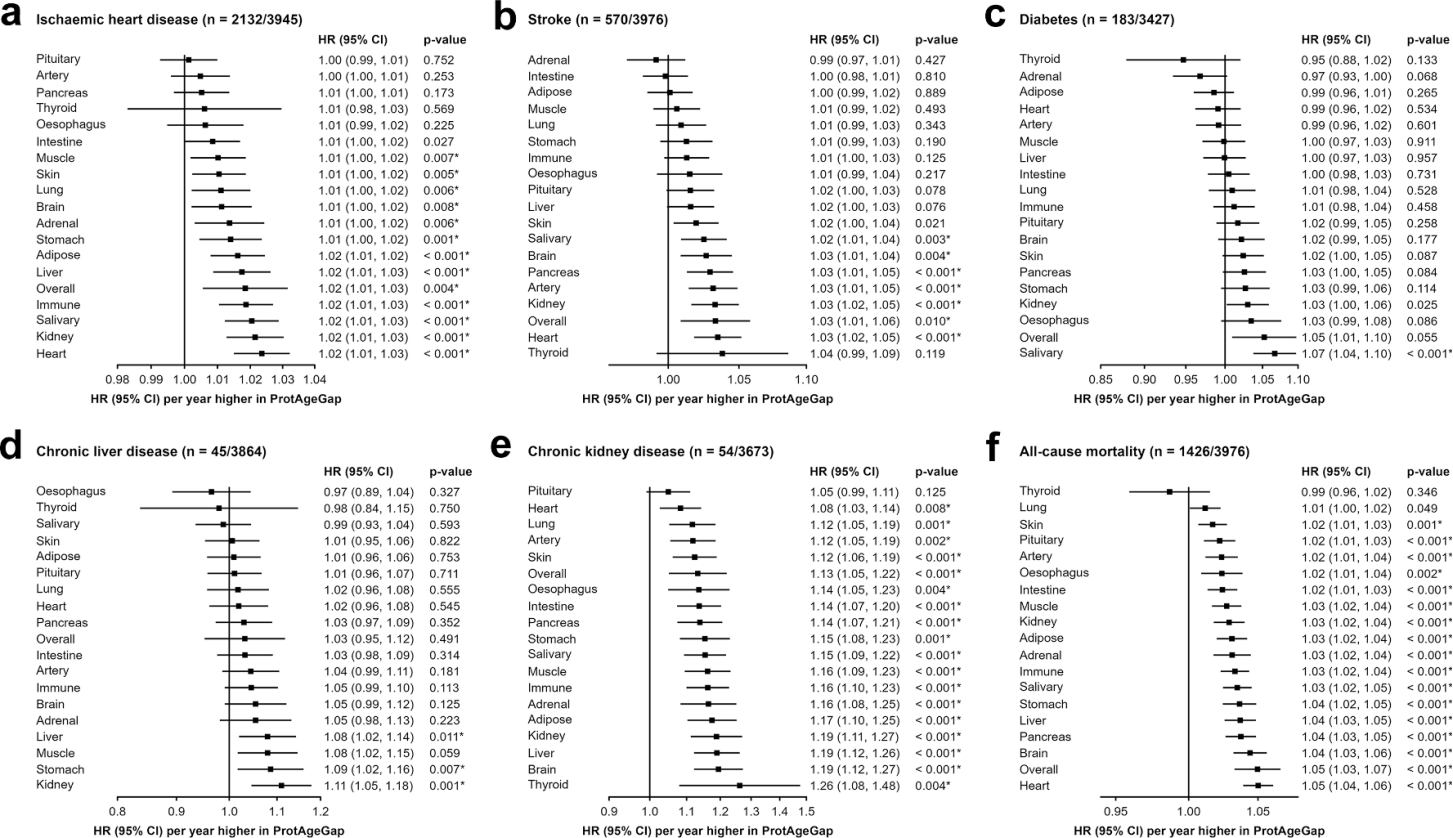
**Associations of overall and organ-specific ProtAgeGaps with key disease endpoints and mortality.** Associations of overall and organ-specific ProtAgeGaps with a. Ischaemic heart disease; b. stroke; c. diabetes; d. chronic liver disease; e. chronic kidney disease; f. all-cause mortality. All ageing clocks were trained based on both Olink and SomaScan proteins after Boruta feature selection. Prentice-weighted (for ischaemic heart disease) and standard (for all other outcomes) Cox regression models were used and adjusted for age, age^2^, sex, region, education level, smoking, alcohol drinking, physical activity, body mass index, prevalent diabetes, and hypertension diagnosis at baseline, where appropriate. Cases of ischaemic heart disease with missing data on development date were excluded from the analysis on Ischaemic heart disease. For analyses of diabetes, chronic liver disease, and chronic kidney disease, participants with the corresponding diseases at baseline were excluded. HR: Hazard ratio. ∗ indicates significant associations after correction for multiple testing with a false discovery rate = 0.05. ProtAgeGap: Difference between proteomic age and chronological age.

The organ-specific and overall ProtAgeGaps were positively associated with a range of major diseases and all-cause mortality (Fig. 2). In particular, the kidney ProtAgeGap was associated with higher risk of chronic kidney disease (CKD, HR = 1.19 [95%CI: 1.11–1.27] per 1-year higher ProtAgeGap); IHD (1.02; 1.01–1.03); stroke (1.03; 1.02–1.05); and chronic liver disease (CLD, 1.11; 1.05–1.18). Overall ProtAgeGap was positively associated with all major diseases except CLD and diabetes (Fig. 2a–e). All of the organ-specific ProtAgeGaps (with the exception of thyroid and lung) and overall ProtAgeGap were statistically significantly and positively associated with higher risk of all-cause mortality (Fig. 2f). Similar patterns were observed albeit with wider confidence intervals when restricting analyses to the subcohort (Supplementary Fig. S11). In sensitivity analyses using models trained specifically on the subcohort only (i.e., excluding IHD cases) we observed broadly similar associations with disease (Supplementary Fig. S12).

Accelerated agers were defined as those >1.5 SD above the mean of age-adjusted ProtAgeGap and participants with organ-specific accelerated ageotypes had higher risk of all-cause mortality for overall and some of the organ-specific ProtAgeGaps except for lung, oesophagus, intestine, pituitary, thyroid and skin (Supplementary Fig. S13). Conversely, super agers were defined as those >1.5 SD below the mean of age-adjusted ProtAgeGap and there was some evidence that super ageotypes of kidney ProtAgeGap was associated with lower risk of stroke and diabetes (Supplementary Fig. S14).

**Fig. 3:**
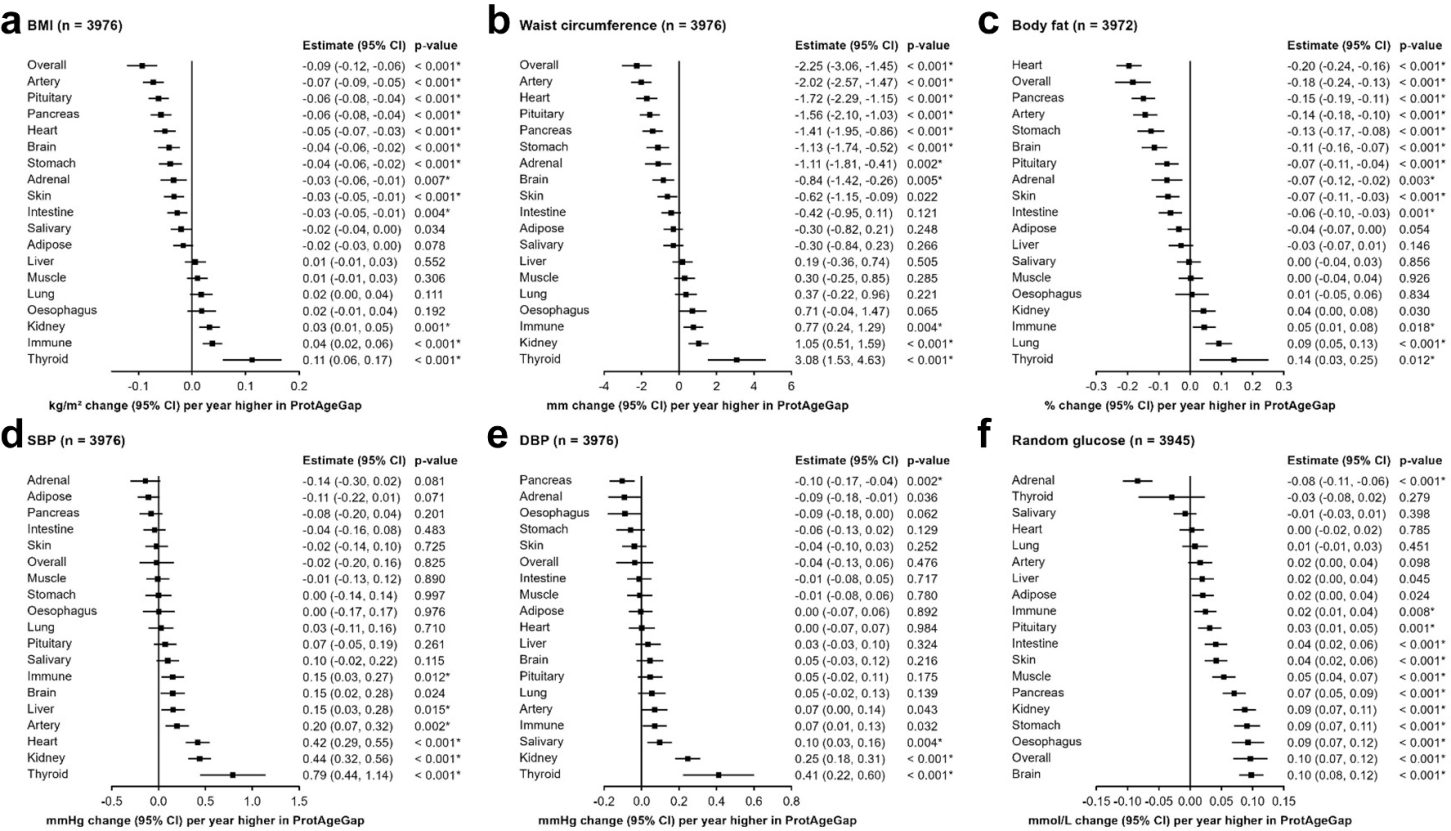
**Associations of overall and organ-specific ProtAgeGaps with physiological measurements at baseline.** Associations of overall and organ-specific ProtAgeGaps with a. BMI; b. waist circumference; c. body fat; d. SBP; e. DBP; f. random glucose. All ageing clocks were trained based on both Olink and SomaScan proteins after Boruta feature selection. All models were adjusted for age, age^2^, sex, region, education level, smoking, alcohol drinking, physical activity, BMI, prevalent diabetes, and hypertension diagnosis at baseline, where appropriate. BMI: Body mass index. SBP: systolic blood pressure. DBP: Diastolic blood pressure. ∗ indicates significant associations after correction for multiple testing with a false discovery rate = 0.05. ProtAgeGap: Difference between proteomic age and chronological age.

Heart, artery, brain and stomach organ-specific ProtAgeGaps were consistently inversely associated with BMI, waist circumference and body fat percentage (Fig. 3a–c). Conversely, the thyroid ProtAgeGap was consistently positively associated with higher BMI, waist circumference and body fat percentage (Fig. 3a–c). The heart, kidney and thyroid ProtAgeGaps had the strongest positive associations with SBP (Fig. 3d), whilst the salivary, kidney and thyroid ProtAgeGaps had the strongest positive associations with DBP (Fig. 3e). There was no evidence that overall ProtAgeGap was associated with SBP or DBP (Fig. 3d and e). Ten of the 18 organ-specific ProtAgeGaps considered in this report were positively associated with random glucose whilst the adrenal ProtAgeGap was inversely associated with random glucose (Fig. 3f). Similar relationships were observed with the organ-specific ProtAgeGaps when the analyses were repeated in the subcohort (Supplementary Fig. S15). In sensitivity analyses that investigated the associations with organ-specific ProtAgeGaps trained on the subcohort data only, the kidney and immune ProtAgeGaps were consistently positively associated with all measures (Supplementary Fig. S16).

When considering participants with organ-specific accelerated ageotypes similar patterns of association were observed with 8 organ-specific ProtAgeGaps being positively associated with random glucose (Supplementary Fig. S17). Considering super ageotypes kidney ProtAgeGap was associated with lower SBP, DBP and random glucose (Supplementary Fig. S18).

**Fig. 4:**
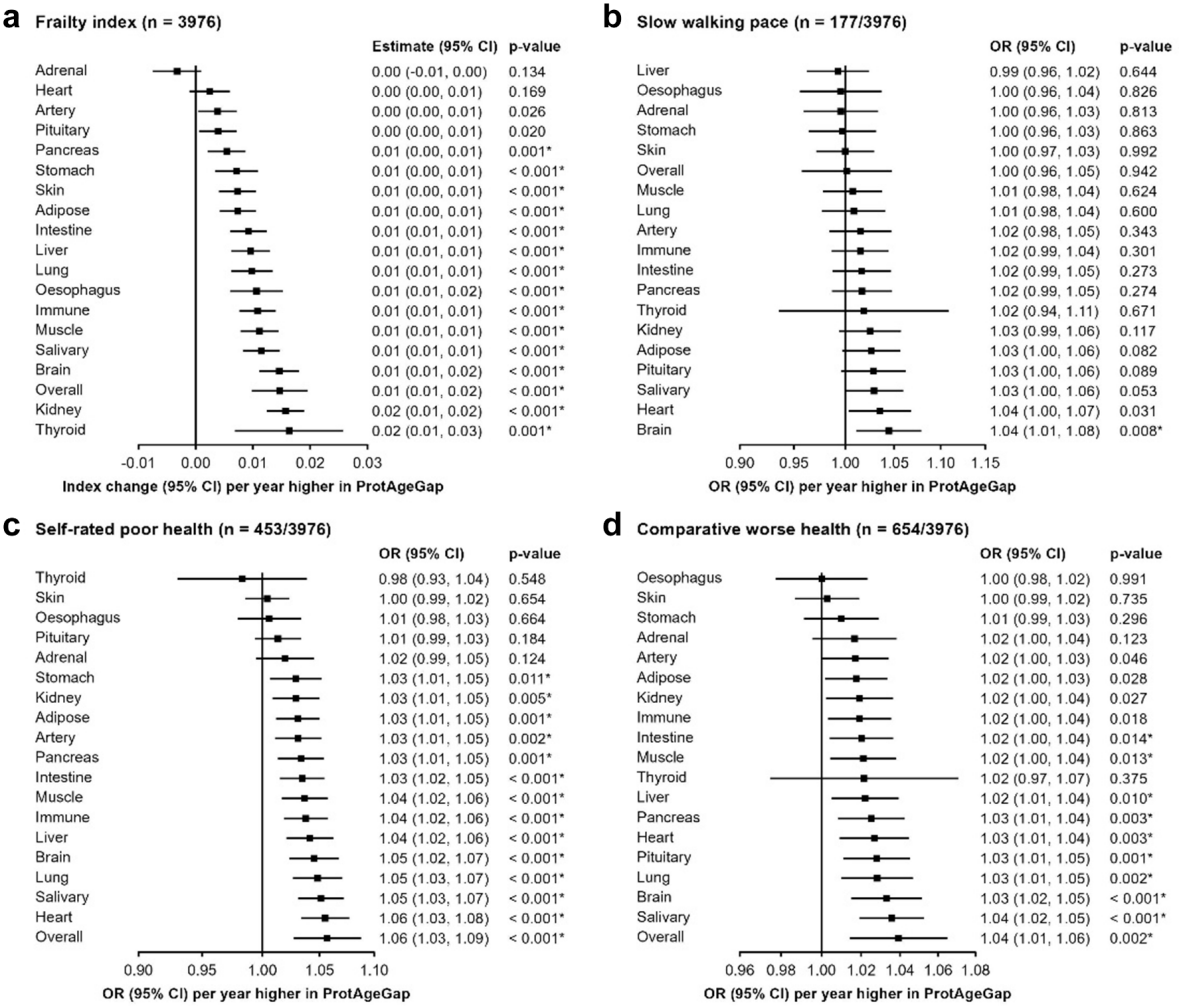
**Associations of overall and organ-specific ProtAgeGaps with ageing-related traits at baseline.** Associations of overall and organ-specific ProtAgeGaps with a. frailty index (scaled by 10 to aid interpretation); b. slow walking pace; c. self-rated poor health; d. comparative worse health. All ageing clocks were trained based on both Olink and SomaScan proteins after Boruta feature selection. All models were adjusted for age, age^2^, sex, region, education level, smoking, alcohol drinking, physical activity, BMI, prevalent diabetes, and hypertension diagnosis at baseline. ∗ indicates significant associations after correction for multiple testing with a false discovery rate = 0.05. ProtAgeGap: Difference between proteomic age and chronological age. ProtAgeGap: Difference between proteomic age and chronological age.

The brain ProtAgeGap was consistently and positively associated with higher frailty index, slow walking pace, self-rated poor health and comparative worse health (Fig. 4a–d). Heart ProtAgeGap was also positively and statistically significantly associated with self-rated poor health and comparative worse health (Fig. 4a–d). Overall ProtAgeGap was associated with frailty index, self-rated poor health and comparative worse health but not slow walking pace (Fig. 4a–d). Patterns and relationships with organ-specific ProtAgeGaps were broadly similar in the subcohort but some associations did not achieve statistical significance due to the smaller number of participants (Supplementary Fig. S19). In sensitivity analyses using ProtAgeGaps trained on the subcohort participants only, the overall, kidney, heart and salivary ProtAgeGaps were positively associated with frailty, self-rated poor health and comparative worse health (Supplementary Fig. S20).

When investigating accelerated ageotypes several organ-specific ProtAgeGaps (including brain, heart, and overall) were mostly positively associated with frailty, self-rated poor health and comparative poor health (Supplementary Fig. S21). For super ageotypes, kidney, muscle and lung ProtAgeGaps were associated with lower frailty index (Supplementary Fig. S22).

For traits measured at resurvey such as hand grip strength and stiffness index there was no clear evidence of any associations for all participants (Supplementary Fig. S23) or in the subcohort participants (Supplementary Fig. S24). Similarly, when investigating accelerated ageotypes there were no clear evidence of any associations (Supplementary Fig. S25) which was also the case for super agers (Supplementary Fig. S26). Results were broadly similar when using proteomic ageing models trained on subcohort participants (Supplementary Fig. S27). Supplementary Fig. S28 summarises the relationships between ProtAgeGaps, age-related diseases and traits considered in this report. The relationship of ProtAgeGaps with other resurvey traits are summarised in Supplementary Fig. S29.

**Fig. 5:**
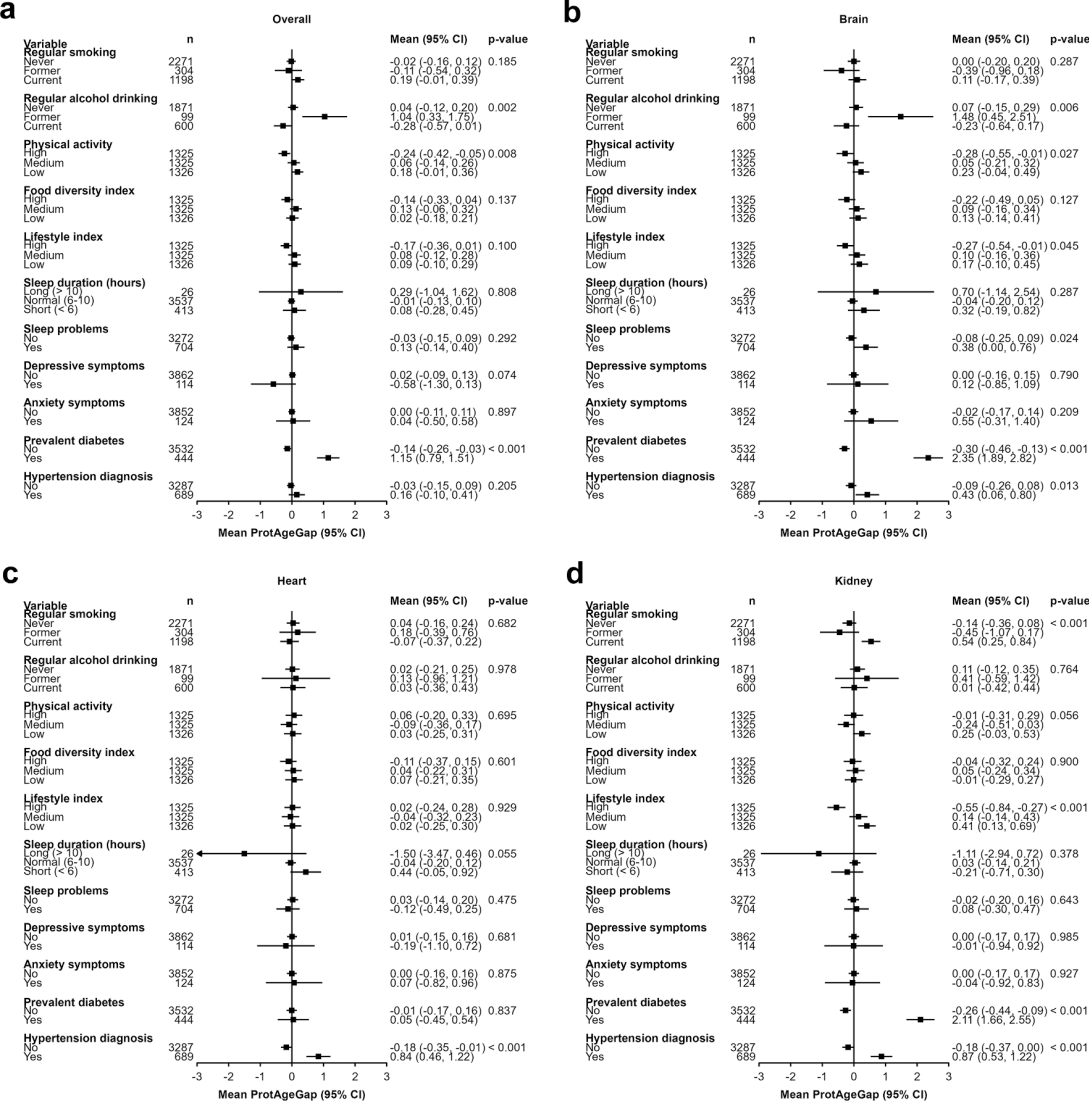
**Overall and key organ-specific ProtAgeGaps by subgroups of modifiable risk factors at baseline.** Overall (a), brain (b), heart (c), and kidney (d) ProtAgeGaps across subgroups of modifiable risk factors at baseline. ProtAgeGap was adjusted for age, age^2^, sex, region, education level, smoking, alcohol drinking, physical activity, BMI, prevalent diabetes, and hypertension diagnosis at baseline, where appropriate. p-values shown here indicate the global p-values from ANOVA F-tests, where each risk factor was the explanatory variable and ProtAgeGap was the outcome. The proteomic ageing clock was trained based on both Olink and SomaScan proteins after Boruta feature selection. Subgroups of physical activity, food diversity index, and lifestyle index were defined based on tertiles. ProtAgeGap: Difference between proteomic age and chronological age.

For the overall ProtAgeGap there was evidence of effect modification by alcohol and diabetes (Fig. 5a). Specifically, former drinkers had a positive ProtAgeGap whilst current drinkers had a negative ProtAgeGap and participants with prevalent diabetes had a positive ProtAgeGap compared to those without prevalent diabetes (Fig. 5a). Moreover, there was some evidence of a trend in physical activity with participants that engaged in high levels of physical activity having a negative ProtAgeGap whilst those with moderate and low levels of physical activity having a positive ProtAgeGap (Fig. 5a). Strong evidence of effect modification by diabetes was also observed for brain ProtAgeGap (Fig. 5b) and kidney ProtAgeGap (Fig. 5d). There was some evidence of a positive heart ProtAgeGap in participants with hypertension compared to those without (Fig. 5c). There was some evidence of effect modification by smoking for kidney ProtAgeGap, with current smokers having a positive ProtAgeGap compared to negative ProtAgeGap for both former and never smokers (Fig. 5d). There was also strong evidence of a trend in kidney ProtAgeGap by lifestyle index with participants with a high healthy lifestyle index having negative ProtAgeGap whilst those with medium and low lifestyle index having positive ProtAgeGap (Fig. 5d).

We assessed the utility of the ProtAgeGaps in predicting the risk of all-cause mortality and IHD events over and above conventional risk factors. For all-cause mortality that included all participants the Heart ProtAgeGap had the largest impact on the C-statistic with a corresponding NRI of 28 (95% CI: 22,35) (Supplementary Table S2). For IHD events that included all participants the Salivary ProtAgeGap had the largest impact on the C-statistic with a corresponding net reclassification index of 18 (95% CI: 12,25) (Supplementary Table S3).

When all-cause mortality analyses were restricted to the subcohort only, the Artery ProtAgeGap had the largest impact on the C-statistic with a corresponding NRI of 53 (95% CI: 38,67) (Supplementary Table S4). Likewise, when the IHD analyses were restricted to the subcohort, Adipose ProtAgeGap had the largest impact on the C-statistic with a corresponding net reclassification index of 39 (95% CI: 26,53) (Supplementary Table S5).

**Fig. 6:**
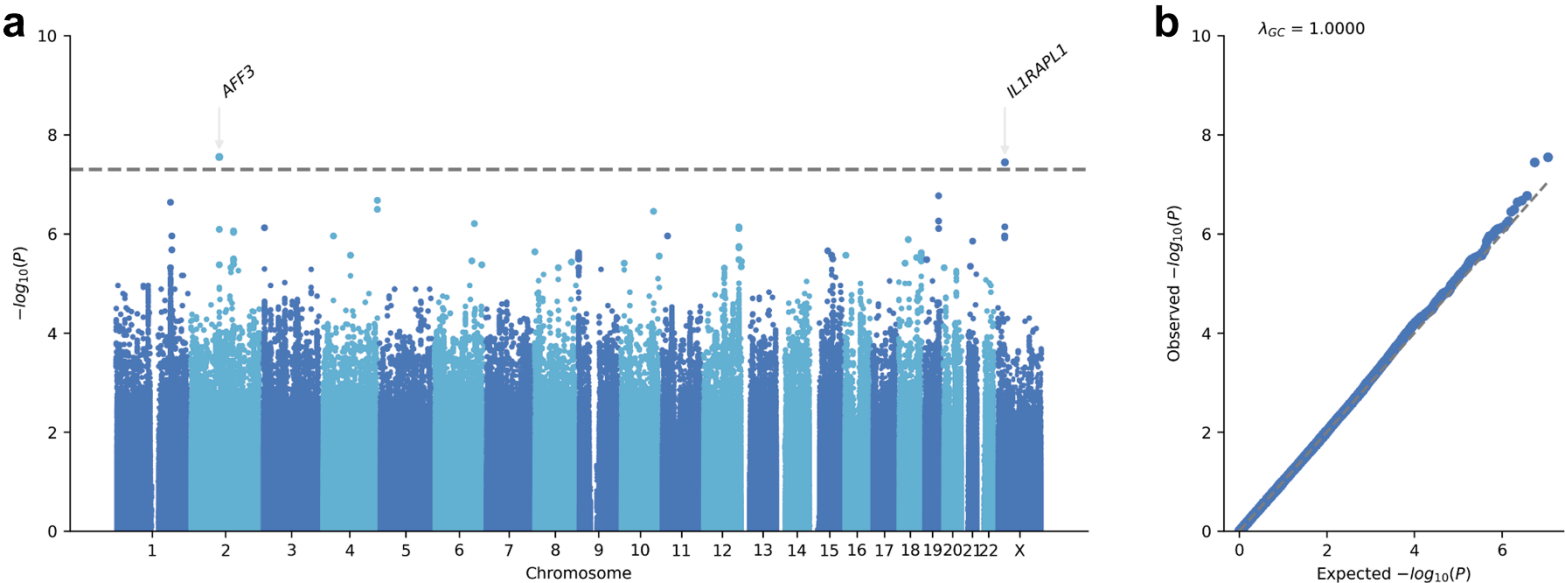
**Manhattan and Q–Q plots of genome-wide associations with overall ProtAgeGap.** a. Manhattan plot. b. Q–Q plot. The analysis was adjusted for age, age^2^, sex, region, and the first 11 genomic principal components. The dotted line indicates the genome-wide significance threshold (5 × 10^−8^). The proteomic ageing clock was trained based on both Olink and SomaScan proteins after Boruta feature selection. Lead variants were identified with a sliding window of 500 kb and annotated with the genes they are located in or next to. ProtAgeGap: Difference between proteomic age and chronological age.

In GWAS of overall proteomic age, we identified two genes (*AFF3 and IL1RAPL1*) that were potentially associated with biological ageing (Fig. 6a) with no evidence of genomic inflation (Fig. 6b). The locus zoom plot shows the linkage disequilibrium coefficients denoted by r^2^ for the region around *AFF3* (Supplementary Fig. S30) and *IL1RAPL1* (Supplementary Fig. S31), respectively.

In individual GWAS for organ-specific ProtAgeGaps we observed GWAS significant results for adipose tissue (ITLN1; Supplementary Fig. S32), adrenal gland (AKR1B1, Supplementary Fig. S33), oesophagus (CST5, Supplementary Fig. S34), immune (HLA-C, Supplementary Fig. S35), intestine (MLN, Supplementary Fig. S36), kidney (HAVCR1 and PDILT, Supplementary Fig. S37), liver (LECT2, Supplementary Fig. S38), lung (MAP3K6, SCGB1A1, CCL18, Supplementary Fig. S39), pancreas (REG1B, PRSS2, Supplementary Fig. S40), pituitary (NPTX2, Supplementary Fig. S41), skin (KALRN, IL21R, CCL13, Supplementary Fig. S42), stomach (TEX2,TMPRSS3, Supplementary Fig. S43). However, we did not identify any GWAS significant results for brain ProtAgeGap (Supplementary Fig. S44). Several of the lead genetic variants identified did not have that protein included in their organ-specific ageing clock (immune [HLA-C], kidney [PDILT], lung [MAP3K6], skin [KALRN, IL21R, CCL13] and stomach [TEX2, TMPRSS3]), which may suggest potential novel pathways (Supplementary Table S6).

## Discussion

This report assessed the relationship of overall and organ-specific ageing with incident diseases and all-cause mortality and our findings suggest that there is only a moderate level of specificity in terms of the relationship with accelerated organ ageing. Despite the positive correlations (0.3–0.9) between the different organ-specific ProtAgeGaps, they were generally weakly correlated with overall ProtAgeGap, suggesting that accelerated ageing in a specific organ can increase the risk of morbidity in other organs, perhaps via cellular ageing.^51^

Our study showed that organ-specific kidney ageing was associated not only with CKD but also with several other conditions (including IHD, CLD, and all-cause mortality) in this East Asian population, consistent with similar findings in Europeans.^11^ The kidneys are known to perform essential functions in the body including waste removal and blood pressure control and thus advanced kidney ageing can be linked to multiple diseases.^52^ As also noted by others, positive ProtAgeGaps in all organ-specific ageing clocks as well as overall proteomic ageing were linked to higher risk of kidney disease.^11^ We also replicated the previous finding that accelerated proteomic heart ageing is associated with IHD and stroke,^10^ and generated new evidence that brain proteomic ageing is also associated with stroke.

All ProtAgeGaps, except the thyroid ProtAgeGap, were associated with all-cause mortality in our study, which persisted even after adjustment for chronological age, prior disease and other risk factors. Several organ-specific ProtAgeGaps (including heart, salivary, liver, immune, brain and kidney), made modest improvements to the prediction of all-cause mortality over conventional risk factors. This suggests that these ProtAgeGaps could be used to identify individuals that may benefit from early interventions that may potentially slow or even reverse the ageing of specific organs and body systems. As a consequence, the overall and organ-specific proteomic ageing clocks, once comprehensively validated, could be used as either predictive, responsive, prognostic or surrogate biomarkers in clinical trials.^51^

We found that the kidney ProtAgeGap was associated with liver disease. It is known that kidney dysfunction affects many organs, including the liver which a complex organ responsible for a considerable part of the metabolism of proteins, fats, and carbohydrates. There is evidence that interaction between the kidney and liver plays a crucial role in normal and certain pathological conditions (e.g., lipid accumulation), and this bidirectional crosstalk may occur via systemic conditions that mutually influence both the liver and kidneys.^53^

The thyroid ProtAgeGap was most strongly associated with kidney disease. It has been reported that the relationship between the thyroid gland and the kidney is mutual, as thyroid hormones are critical regulators of kidney structure and function. There is mounting evidence that subclinical hypothyroidism is more common in people with CKD than in the general population, and could be a potential risk factor for the development of CKD.^54^ Similarly, the thyroid ProtAgeGap was most strongly associated with several intermediate cardiometabolic measures. Hypothyroidism, which is characterised by reduced thyroid hormone production, is known to primarily associated with metabolic and endocrine alterations, hypothyroidism has also been associated with an increased risk for cardiovascular disease (CVD), mainly due to accelerated atherosclerosis.^55^

We demonstrated that ProtAgeGaps were associated with several modifiable risk factors (e.g., SBP, BMI and random glucose). Kidney ProtAgeGap was positively associated with a range of risk factors and traits. However, brain ProtAgeGap was also found to be inversely associated with BMI and body fat percentage whilst positively associated with SBP, random glucose in addition to age-related traits such as frailty and slow walking pace. There is evidence that modifiable lifestyle factors such as adiposity and sleep duration are causally related to multiple organ-specific age gaps.^56^

We performed GWAS to better understand the genetic architecture of these ProtAgeGaps and identified genetic variants in ALF Transcription Elongation Factor 3 (*AFF3*) and Interleukin 1 Receptor Accessory Protein Like 1 (*IL1RAPL1*) genes associated with overall ProtAgeGap. These two proteins were not included in the overall ageing clock suggesting they may have a pervasive impact in ageing. The *AFF3* gene encodes a tissue-restricted nuclear transcriptional activator that is preferentially expressed in lymphoid tissue. Isolation of this protein initially defined a highly conserved AF4/FMR2 family of nuclear transcription factors that may function in lymphoid development and oncogenesis and may also play a role in rheumatoid arthritis and type 1 diabetes.^57^ *IL1RAPL1* is a protein coding gene and the protein encoded by this gene is a member of the interleukin 1 receptor family and is similar to the interleukin 1 accessory proteins.^58^ However, as this gene was not in linkage disequilibrium with nearby variants this association may not be robust. Our GWAS findings for overall ProtAgeGap using a combination of affinity-based proteomic platforms differ from those reported by Wang et al. in Europeans that utilised proteins from the Olink platform only.^59^ In that study, the top five ranking genes were *KLHL22, MED15, SCARF2, ZNF74,* and *SMAD5*, that are known to have roles in ageing and age-related diseases.^59^ The differences are mostly likely due to the combination of Olink and SomaScan platforms and the smaller sample size available in CKB. Wang et all. noted about half of the proteins in their Olink-only based clocks were not identified in SomaScan-only based clocks.^59^

Our GWAS also identified a range of known variants associated with selected organ-specific ageing clocks that contained the protein, including *ITLN1* for the adipose proteomic ageing, which is also known as Intelectin 1, a protein coding gene that plays and important role in glucose metabolism,^60^ and is known to be associated with obesity related diseases.^61^ Protein Disulfide Isomerase-Like Protein of the Testis (PDILT), which encodes a member of the disulfide isomerase (PDI) family of endoplasmic reticulum (ER) proteins that catalyse protein folding and thiol-disulfide interchange reactions, was associated with kidney ProtAgeGap in GWAS analyses and was not included in the kidney organ-ageing clock, although its nearby gene UMOD was included. GWAS studies of CKD progression have identified the *UMOD/PDILT* region as important in multi-ancestry populations. Specifically, for rs77924615, near *UMOD/PDILT*, each copy of the G allele was associated with a 0.30%/year faster eGFR decline.^62^ In contrast to that reported by others^63^ we found no GWAS significant associations with brain proteomic ageing in this East Asian population which is most likely due to lack of statistical power. However, we acknowledge that genetic factors may not be the main drivers of biological ageing. For example, it was recently reported that non-genetic exposures were more strongly associated with age-related diseases than genetics or previously developed overall proteomic ageing clocks in the UK Biobank.^64^ Their results suggest that the exposome is much more influential on patterns of disease and mortality, irrespective of polygenic disease risk.^64^

Apart from leveraging an East Asian population, the main strengths of this study include a large number of proteins assayed using two affinity-based technologies, the wide range of age-related traits and diseases considered, and the GWAS analyses to understand the genetic architecture of the overall and organ-specific ageing clocks. The combination of Olink and SomaScan platforms to develop overall and organ-specific proteomic ageing clocks appears to add value over other published overall proteomic ageing clocks based on a single assay platform.^8^^,^^10^^,^^63^ However, the study also had several limitations. First, we used chronological age to train our proteomic ageing clocks and then computed the ProtAgeGap overall and for 18 organs separately. Others have used a combination of proteins and chronological age at baseline to train a LASSO penalised Cox regression model for the risk of all-cause mortality. The selected proteins and chronological age were then used to fit Gompertz proportional hazards models and develop the proteomic ageing clock to estimate the proteomic age based on these input data.^65^ Ageing clocks trained on chronological age are susceptible to the biomarker paradox whereby an absolutely precise clock (i.e., estimates chronological age perfectly), would not be helpful for health assessment.^66^ Moreover, our overall and organ-specific proteomic ageing clocks trained on chronological age may give different results to proteomic ageing clocks trained on mortality. However, our overall ProtAgeGap (trained on chronological age) included overlapping proteins to the proteomic ageing clock (trained on mortality), that were representative of hallmarks of biological ageing, including immunoinflammatory responses, cellular senescence, extracellular matrix remodelling, cellular response to stressors, and vascular biology.^65^

Second, we based our analytic strategy on our previous publications which used all participants in the China Kadoorie Biobank proteomics case-cohort study^8^^,^^67^ to maximise statistical power, but this may bias the results. We investigated the extent of the potential bias caused by the inclusion of IHD cases by conducting separate analyses using overall and organ-specific ProtAge trained on the subcohort participants, which showed consistent results with the main analyses. Third, we based our ProtAgeGaps on the difference between estimated overall and organ-specific proteomic ageing and chronological age, which may lead to systematic bias, whereby the predicted biological age may be overestimated for younger participants and underestimated for older participants. We endeavoured to mitigate against this bias by conducting analyses whereby we regressed each of our overall and organ-specific ProtAge estimates on chronological age and defined the ProtAgeGaps using the residuals from each of these respective regressions. We repeated our analyses of diseases and age-related phenotypes using this alternative definition of ProtAgeGaps based on residuals and the results were broadly consistent (Supplementary Figs. S45–S60). Fourth, although it is the largest proteomics study to date in East Asians, power was limited so we only considered selected diseases for investigation. Nonetheless, our findings provide valuable insights particularly for all-cause mortality and CKD. Fifth, the cross-sectional study design and lack of repeated measurements of proteins over time prevent confirmation of the direction of observed associations. Sixth, despite extensive adjustment for key covariates to minimise confounding, residual confounding may still be present. Finally, the lack of similar datasets with proteomics data prevented the full replication of our findings in independent East Asian cohorts. However, the findings in the present study are also directionally and broadly consistent with published organ-specific and overall ageing clocks using Olink or SomaScan proteins.^10^^,^^11^

In summary, the present study in Chinese adults demonstrated that overall and organ-specific proteomic ageing clocks developed using two complementary affinity-based platforms were related to age-related traits and diseases. Future larger studies from diverse cohorts are still required to replicate our findings and to identify new associations, which may guide the development of surrogate endpoints or ageing biomarker discovery. Further exploration of the genetic variants associated with overall and organ-specific proteomic ageing clocks via Mendelian Randomisation and colocalisation analyses, may identify the causal relevance of these associations. The findings of this study provide further evidence that proteomic ageing is driven by multiple genetic and environmental determinants and is potentially modifiable by lifestyle and behavioural changes. Additional downstream analyses and functional validation are needed to confirm and elucidate biological mechanisms behind proteomic ageing to identify possible drug targets or drug repurposing opportunities for geroprotection.

## Contributors

DAB, BW, ZC conceived and designed the study. BW, NW, AE, SX accessed and verified the underlying data and conducted the statistical analyses, DAB and BW wrote the first draft of the manuscript. ZC and LL as members of China Kadoorie Biobank Steering Committee, designed and supervised the overall conduct of the study, including obtaining funding for the study. All other authors provided critical revision to the manuscript for important intellectual content. DAB, BW and ZC are the guarantors of this work and take responsibility for the integrity and accuracy of the data analysis. DAB and ZC supervised the work. All named authors read and approved the final version of the manuscript. Members of the China Kadoorie Biobank Collaborative Group contributed to the overall conduct of the study and collection of data utilised in this report.

## Data sharing statement

In CKB, non-genetic data (e.g., baseline, resurveys, biomarkers, and disease follow-up) are released periodically to bona fide researchers. Details of the CKB Data Sharing Policy, data release schedules and data request application procedures are available at www.ckbiobank.org. All queries about data access can be made to ckbaccess@ndph.ox.ac.uk. The code used to conduct these analyses is available here: https://github.com/ckbiobank/Bennett-Wang-2026-proteomic-ageing-clocks.

## Declaration of interests

All authors declare no competing interests.
