# Supplementary material for: Proteomic organ-specific signatures, ageing traits, disease risks and genetic architecture in an East Asian population
Source: eBioMedicine. 2026 Jul 29;130:106401. doi: 10.1016/j.ebiom.2026.106401 (PMC13450583; doi:10.1016/j.ebiom.2026.106401)
Supplement: Supplementary file 1 [file mmc2.docx]

Contents

[Members of the China Kadoorie Biobank Collaborative Group	5](#_Toc234057523)

[Supplementary Methods	6](#_Toc234057524)

[Supplementary Figure 1. Case-cohort study design.	8](#_Toc234057525)

[Supplementary Figure 2. Overlap of proteins between the overall ageing clocks.	9](#_Toc234057526)

[Supplementary Figure 3. Overlap of proteins included in the overall ageing clocks between the current and previous studies.	10](#_Toc234057527)

[Supplementary Figure 4. Performance of overall ageing clocks in holdout test data, trained based on all, FDR-significant, and Boruta-selected proteins.	11](#_Toc234057528)

[Supplementary Figure 5. Performance of overall ageing clocks in holdout test data in subcohort participants, trained based on all, FDR-significant, and Boruta-selected proteins.	12](#_Toc234057529)

[Supplementary Figure 6. Distribution of overall ProtAgeGap by socio-demographic groups.	13](#_Toc234057530)

[Supplementary Figure 7. Distribution of overall ProtAgeGap by socio-demographic groups in subcohort participants.	14](#_Toc234057531)

[Supplementary Figure 8. Performance and pairwise correlations of overall and organ-specific ageing clocks in the holdout test set.	15](#_Toc234057532)

[Supplementary Figure 9. Performance and pairwise correlations of overall and organ-specific ageing clocks in the holdout test set in subcohort participants.	16](#_Toc234057533)

[Supplementary Figure 10. Performance and pairwise correlations of overall and organ-specific ageing clocks in the UK Biobank.	17](#_Toc234057534)

[Supplementary Figure 11. Associations of overall and organ-specific ProtAgeGaps with disease endpoints and mortality in subcohort participants.	18](#_Toc234057535)

[Supplementary Figure 12. Associations of overall and organ-specific ProtAgeGaps with disease endpoints and mortality using models trained in subcohort participants.	19](#_Toc234057536)

[Supplementary Figure 13. Associations of overall and organ-specific accelerated ageotypes with disease endpoints and mortality.	20](#_Toc234057537)

[Supplementary Figure 14. Associations of overall and organ-specific super ageotypes with disease endpoints and mortality.	21](#_Toc234057538)

[Supplementary Figure 15. Associations of overall and organ-specific ProtAgeGaps with physiological measurements at baseline in subcohort participants.	22](#_Toc234057539)

[Supplementary Figure 16. Associations of overall and organ-specific ProtAgeGaps with physiological measurements at baseline using models trained in subcohort participants.	23](#_Toc234057540)

[Supplementary Figure 17. Associations of overall and organ-specific accelerated ageotypes with physiological measurements at baseline.	24](#_Toc234057541)

[Supplementary Figure 18. Associations of overall and organ-specific super ageotypes with physiological measurements at baseline.	25](#_Toc234057542)

[Supplementary Figure 19. Associations of overall and organ-specific ProtAgeGaps with ageing-related traits at baseline in subcohort participants.	26](#_Toc234057543)

[Supplementary Figure 20. Associations of overall and organ-specific ProtAgeGaps with ageing-related traits at baseline using models trained in subcohort participants.	27](#_Toc234057544)

[Supplementary Figure 21. Associations of overall and organ-specific accelerated ageotypes with ageing-related traits at baseline.	28](#_Toc234057545)

[Supplementary Figure 22. Associations of overall and organ-specific super ageotypes with ageing-related traits at baseline.	29](#_Toc234057546)

[Supplementary Figure 23. Associations of overall and organ-specific ProtAgeGaps with resurvey characteristics.	30](#_Toc234057547)

[Supplementary Figure 24. Associations of overall and organ-specific ProtAgeGaps with resurvey characteristics in subcohort participants.	31](#_Toc234057548)

[Supplementary Figure 25. Associations of overall and organ-specific accelerated ageotypes with resurvey characteristics.	32](#_Toc234057549)

[Supplementary Figure 26. Associations of overall and organ-specific super ageotypes with resurvey characteristics.	33](#_Toc234057550)

[Supplementary Figure 27. Associations of overall and organ-specific ProtAgeGaps with resurvey characteristics using models trained in subcohort participants.	34](#_Toc234057551)

[Supplementary Figure 28. Relationships between ProtAgeGaps and age-related diseases and traits.	35](#_Toc234057552)

[Supplementary Figure 29. Relationships between ProtAgeGaps and resurvey traits.	36](#_Toc234057553)

[Supplementary Figure 30. Regional plot of the locus at AFF3 associated with overall ProtAgeGap.	37](#_Toc234057554)

[Supplementary Figure 31. Regional plot of the locus near IL1RAPL1 associated with overall ProtAgeGap.	38](#_Toc234057555)

[Supplementary Figure 32. Manhattan and Q-Q plots of genome-wide associations with adipose ProtAgeGap.	39](#_Toc234057556)

[Supplementary Figure 33. Manhattan and Q-Q plots of genome-wide associations with adrenal ProtAgeGap.	40](#_Toc234057557)

[Supplementary Figure 34. Manhattan and Q-Q plots of genome-wide associations with oesophagus ProtAgeGap.	41](#_Toc234057558)

[Supplementary Figure 35. Manhattan and Q-Q plots of genome-wide associations with immune ProtAgeGap.	42](#_Toc234057559)

[Supplementary Figure 36. Manhattan and Q-Q plots of genome-wide associations with intestine ProtAgeGap.	43](#_Toc234057560)

[Supplementary Figure 37. Manhattan and Q-Q plots of genome-wide associations with kidney ProtAgeGap.	44](#_Toc234057561)

[Supplementary Figure 38. Manhattan and Q-Q plots of genome-wide associations with liver ProtAgeGap.	45](#_Toc234057562)

[Supplementary Figure 39. Manhattan and Q-Q plots of genome-wide associations with lung ProtAgeGap.	46](#_Toc234057563)

[Supplementary Figure 40. Manhattan and Q-Q plots of genome-wide associations with pancreas ProtAgeGap.	47](#_Toc234057564)

[Supplementary Figure 41. Manhattan and Q-Q plots of genome-wide associations with pituitary ProtAgeGap.	48](#_Toc234057565)

[Supplementary Figure 42. Manhattan and Q-Q plots of genome-wide associations with skin ProtAgeGap.	49](#_Toc234057566)

[Supplementary Figure 43. Manhattan and Q-Q plots of genome-wide associations with stomach ProtAgeGap.	50](#_Toc234057567)

[Supplementary Figure 44. Manhattan and Q-Q plots of genome-wide associations with brain ProtAgeGap.	51](#_Toc234057568)

[Supplementary Figure 45. Associations of overall and organ-specific ProtAgeGaps (Residuals) with disease endpoints and mortality using models trained in all participants and applied to all participants.	52](#_Toc234057569)

[Supplementary Figure 46. Associations of overall and organ-specific ProtAgeGaps (Residuals) with physiological measurements at baseline trained in all participants and applied to all participants.	53](#_Toc234057570)

[Supplementary Figure 47. Associations of overall and organ-specific ProtAgeGaps (Residuals) with ageing-related traits at baseline using models trained in all participants and applied to all participants.	54](#_Toc234057571)

[Supplementary Figure 48. Associations of overall and organ-specific ProtAgeGaps (Residuals) with resurvey characteristics using models trained in all participants and applied to all participants.	55](#_Toc234057572)

[Supplementary Figure 49. Associations of overall and organ-specific ProtAgeGaps (Residuals) with disease endpoints and mortality using models trained in all participants and applied to subcohort participants.	56](#_Toc234057573)

[Supplementary Figure 50. Associations of overall and organ-specific ProtAgeGaps (Residuals) with physiological measurements at baseline trained in all participants and applied to subcohort participants.	57](#_Toc234057574)

[Supplementary Figure 51. Associations of overall and organ-specific ProtAgeGaps (Residuals) with ageing-related traits at baseline using models trained in all participants and applied to subcohort participants.	58](#_Toc234057575)

[Supplementary Figure 52. Associations of overall and organ-specific ProtAgeGaps (Residuals) with resurvey characteristics using models trained in all participants and applied to subcohort participants.	59](#_Toc234057576)

[Supplementary Figure 53. Associations of overall and organ-specific ProtAgeGaps (Residuals) with disease endpoints and mortality using models trained in subcohort participants and applied to all participants.	60](#_Toc234057577)

[Supplementary Figure 54. Associations of overall and organ-specific ProtAgeGaps (Residuals) with physiological measurements at baseline trained in subcohort participants and applied to all participants.	61](#_Toc234057578)

[Supplementary Figure 55. Associations of overall and organ-specific ProtAgeGaps (Residuals) with ageing-related traits at baseline using models trained in subcohort participants and applied to all participants.	62](#_Toc234057579)

[Supplementary Figure 56. Associations of overall and organ-specific ProtAgeGaps (Residuals) with resurvey characteristics using models trained in subcohort participants and applied to all participants.	63](#_Toc234057580)

[Supplementary Figure 57. Associations of overall and organ-specific ProtAgeGaps (Residuals) with disease endpoints and mortality using models trained in subcohort participants and applied to subcohort participants.	64](#_Toc234057581)

[Supplementary Figure 58. Associations of overall and organ-specific ProtAgeGaps (Residuals) with physiological measurements at baseline trained in subcohort participants and applied to subcohort participants.	65](#_Toc234057582)

[Supplementary Figure 59. Associations of overall and organ-specific ProtAgeGaps (Residuals) with ageing-related traits at baseline using models trained in subcohort participants and applied to subcohort participants.	66](#_Toc234057583)

[Supplementary Figure 60. Associations of overall and organ-specific ProtAgeGaps (Residuals) with resurvey characteristics using models trained in subcohort participants and applied to subcohort participants.	67](#_Toc234057584)

[Supplementary Table 1. Characteristics of study participants at baseline and resurvey by case-subcohort status.	68](#_Toc234057585)

[Supplementary Table 2. Performance of Cox risk prediction models for all-cause mortality using conventional risk factors and overall and organ-specific ProtAgeGaps, assessed by C-statistics and Net Reclassification Index (all participants).	70](#_Toc234057586)

[Supplementary Table 3. Performance of Cox risk prediction models for ischaemic heart disease using conventional risk factors and overall and organ-specific ProtAgeGaps, assessed by C-statistics and Net Reclassification Index (all participants).	71](#_Toc234057587)

[Supplementary Table 4. Performance of Cox risk prediction models for all-cause mortality using conventional risk factors and overall and organ-specific ProtAgeGaps, assessed by C-statistics and Net Reclassification Index (subcohort only).	72](#_Toc234057588)

[Supplementary Table 5. Performance of Cox risk prediction models for ischaemic heart disease using conventional risk factors and overall and organ-specific ProtAgeGaps, assessed by C-statistics and Net Reclassification Index (subcohort only).	73](#_Toc234057589)

[Supplementary Table 6. Summary of lead variants from GWAS of overall and organ-specific ProtAgeGaps.	74](#_Toc234057590)

**Members of the China Kadoorie Biobank Collaborative Group**

**International Steering Committee:** Junshi Chen, Zhengming Chen (PI), Rory Collins, Liming Li (PI), Jun Lv, Richard Peto, Robin Walters, Canqing Yu, Dianjianyi Sun and Derrick Bennett.

**International Co-ordinating Centre, Oxford:** Daniel Avery, Maxim Barnard, Derrick Bennett, Ruth Boxall, Ka Hung Chan, Yiping Chen, Zhengming Chen, Jonathan Clarke, Huaidong Du, Ahmed Edris Mohamed, Hannah Fry, Yani Huang, Pek Kei Im, Andri Iona, Christiana Kartsonaki, Kshitij Kolhe, Hubert Lam, Kuang Lin, Iona Millwood, Sam Morris, Qunhua Nie, Sara Perez Vizan, Thu Pham, Alfred Pozarickij, Maryam Rahmati, Paul Ryder, Maruf Sarder, Dan Schmidt, Roshi Shrestha, Becky Stevens, Robin Walters, Lin Wang, Neil Wright, Ling Yang, Xiaoming Yang, Pang Yao.

**National Co-ordinating Centre, Beijing:** Liming Li, Jun Lv, Canqing Yu, Dianjianyi Sun, Yuanjie Pang, Yuting Han, Can Hou, Qingmei Xia, Chao Liu, Pei Pei, Lang Pan, Xiao Han, Honglu Bian, Xinxin Chen.

**10 Regional Co-ordinating Centres:**

**Qingdao CDC:** Zengchang Pang, Ruqin Gao, Kunzheng Lv, Shanpeng Li, Haiping Duan, Shaojie Wang, Yongmei Liu, Ranran Du, Liang Cheng, Xiaocao Tian, Hua Zhang. **Licang CDC:** Dan Hu, Xiaoyan Zheng, Yujie Wang. **Heilongjiang Provincial CDC:** Wei Sun, Shichun Yan, Yong Zhou. **Nangang CDC:** Chi Wang, Zhenyuan Wu, Lishun Zhai, Zhaoxi Pang, Shiwen Dong, Li Liu. **Hainan Provincial CDC:** Dapeng Yin, Bin He, Ying Liu, Xingren Wang, Tingting Ou. **Meilan CDC:** Xiangyang Zheng, Dewei Zheng, Shuai Yang, Lihui Li, Xingjiao Chen. **Jiangsu Provincial CDC:** Yan Xu, Jinyi Zhou, Ran Tao, Jian Su, Xikang Fan, Xuejia Chen, Yuxiao Huang. **Suzhou CDC:** Yan Lu, Yujie Hua, Li Xing, Shuxian Wang, Jianrong Jin, Juping Ma, Jinchao Liu, Kaifei Zhu, Hongfu Ren, Xingfeng Shen. **Guangxi Provincial CDC:** Ge Zhong, Wei Mao, Zhenzhen Lu, Yanxu Zhong. **Liuzhou CDC:** Lifang Zhou, Rong Pan, Jian Lan, Xiaoping Tan, Jinxue Tan, Yishan Xie, Liuping Wei, Liyuan Zhou, Sisi Wang. **Sichuan Provincial CDC:** Xianping Wu, Ningmei Zhang, Xiaofang Chen, Xiaoyu Chang, Zhuo Wang, Yujin He. **Pengzhou CDC:** Mingqiang Yuan, Ling Wang, Xiaofang Chen, Zhaodong Wang, Qiang Sun, Yang Lin. **Gansu Provincial CDC:** Faqing Chen, Xiaolan Ren, Lijun Chang, Feiming Zhong. **Maiji CDC:** Jianjun Feng, Weijie Hu, Xiaofang Zhang, Yalin Chen, Honghong Wang, Jun Wang. **Henan Provincial CDC:** Linqi Diao, Zhiwei Han, Dengjun Zhu, Kai Kang, Shixian Feng, Wenjie Yang, Huizi Tian, Yali Yan, Bing Han, Li Gao, Shaofang Li, Tianfang Xing, Wei Tang, Lei Fan. **Huixian CDC:** Xiaolin Li, Huarong Sun, Xiaocong Zhao, Ying Li, Chen Hu, Pan He, Xukui Zhang, Yuanyuan Jin, Lan Luo. **Zhejiang Provincial CDC:** Min Yu, Ruying Hu, Hao Wang, Weiwei Gong, Jieming Zhong, Meng Wang, Chunxiao Xu, Keqing Gong. **Tongxiang CDC:** Hao Xu, Yuan Cao, Kaixu Xie, Lingli Chen, Xiaomei Tu, Junlong Pan. **Hunan Provincial CDC:** Xiaojun Li, Li Yin, Huilin Liu, Yuan Liu, Lei Yin, Xian Xie, Jing Wang. **Liuyang CDC:** Bo Xiao, Zongwei Deng, Yuan Peng, Libo Zhang, Chan Qu, li Deng, Qili Jiang, Yanling Chen.

# Supplementary Methods

Genotyping of CKB participants was previously described.^1^ In brief, blood samples were obtained at baseline, and DNA extraction and genotyping were performed at BGI, Shenzhen, China. Custom-designed arrays on the Affymetrix (now Thermo Fisher Scientific) Axiom platform were used for genotyping. Raw genotyping data were exported from China to the Oxford CKB International Coordinating Centre under Data Export Approvals 2014-13 and 2015-39 from the Office of Chinese Human Genetic Resource Administration. Data processing, genotype calling, and quality control were conducted at CTSU, University of Oxford, UK. Genotyping calling was performed using GenomeStudio software, with initial QC based on automated clustering. After excluding duplicate samples and samples with call rate <95%, excess heterozygosity, excess homozygosity, sex mismatch, XY aneuploidy, or non-Chinese ancestry (> 10 SDs deviation from the CKB-wide mean for at least one of the first 10 PCs), 100,706 samples passed QC. Imputation was conducted as follows: genotypes of 531,565 variants passing QC in all 100,706 genotyped samples were converted to genome build 38 using CrossMap v0.6.165 and were checked for consistency by reversing the process (“liftUnder”). Probesets with an HWE p < 10−6 (10 degree of freedom sum-of-Chi-squared test) were excluded. Variants not mapped, mapped to different chromosomes, or not mapped back to the same locations after liftUnder, were excluded. The remaining 531,542 variants were prephased using SHAPEIT v4.2^2^ (SHAPEIT v2.904 for chromosome X), and genotypes were imputed using (i) the TOPMed imputation server (release 2 v1.0.0)  and (ii) the Westlake Biobank for Chinese imputation server; the two sets of imputed data were merged, for each variant retaining the imputed genotypes with the higher imputation INFO score. Variants with INFO<0.3 or MAF=0 were excluded, giving a final imputed dataset of 17,933,159 variants with MAF>0.001.

# Supplementary Figure 1. Case-cohort study design.

Note. *19 individuals included in both MI and fatal non-MI IHD cases. **29 individuals included in both IHD cases and subcohort. CVD: Cardiovascular disease. MI: Myocardial infarction. IHD: Ischaemic heart disease. GWAS: Genome-wide association studies. QC: Quality control.

# Supplementary Figure 2. Overlap of proteins between the overall ageing clocks.

Note. a. Overlap between all Olink and all SomaScan proteins. b. Overlap between FDR-significant Olink and SomaScan proteins. c. Overlap between Boruta-selected Olink and SomaScan proteins. ‘Both’ in indicates Boruta-selected proteins in the overall ageing clock trained based on both Olink and SomaScan proteins, while ‘Olink’ and ‘SomaScan’ indicate models trained based on proteins from a single platform. Numbers shown here were estimated based on UniProt IDs to allow direct comparison between platforms. Some SomaScan reagents correspond to more than one UniProt IDs.

# Supplementary Figure 3. Overlap of proteins included in the overall ageing clocks between the current and previous studies.

Note. a. Overlap of Olink proteins (ageing clock in the current study corresponds to the one trained based on both Olink and SomaScan proteins). b. Overlap of SomaScan proteins (ageing clock in the current study corresponds to the one trained based on both Olink and SomaScan proteins). c. Overlap of Olink proteins (ageing clock in the current study corresponds to the one trained based on Olink only). d. Overlap of SomaScan proteins (ageing clock in the current study corresponds to the one trained based on SomaScan only). Proteins in the current study and Argentieri et al. (2024) were selected by Boruta. Proteins in Oh et al. (2023) were selected by LASSO regression.

# Supplementary Figure 4. Performance of overall ageing clocks in holdout test data, trained based on all, FDR-significant, and Boruta-selected proteins.

Note. Performance of overall ageing clocks in the holdout test data (n = 1,193), trained based on a. Olink proteins only; b. SomaScan proteins only; c. both Olink and SomaScan proteins. FDR: Proteins significantly associated with age, after correction for multiple testing with a false discovery rate = 0.05. ProtAge: Proteomic Age. ChronAge: Chronological age at recruitment.

# Supplementary Figure 5. Performance of overall ageing clocks in holdout test data in subcohort participants, trained based on all, FDR-significant, and Boruta-selected proteins.

Note. Performance of overall ageing clocks in the holdout test data in subcohort participants (n = 608), trained based on a. Olink proteins only; b. SomaScan proteins only; c. both Olink and SomaScan proteins. FDR: Proteins significantly associated with age, after correction for multiple testing with a false discovery rate = 0.05. ProtAge: Proteomic Age. ChronAge: Chronological age at recruitment.

# Supplementary Figure 6. Distribution of overall ProtAgeGap by socio-demographic groups.

Note. Distribution of overall ProtAgeGap by socio-demographic group in all participants (n = 3,976), with ageing clock models trained based on a. Olink proteins only; b. SomaScan proteins only; c. both Olink and SomaScan proteins. The models were trained based on proteins after Boruta feature selection. ProtAgeGap was adjusted for chronological age, age^2^, sex, and study region, unless the variable was plotted as the x-axis. ProtAgeGap: Difference between proteomic age and chronological age.

# Supplementary Figure 7. Distribution of overall ProtAgeGap by socio-demographic groups in subcohort participants.

Note. Distribution of overall ProtAgeGap by socio-demographic group in subcohort participants (n = 2,025), with ageing clock models trained based on a. Olink proteins only; b. SomaScan proteins only; c. both Olink and SomaScan proteins. The models were trained based on proteins after Boruta feature selection. ProtAgeGap was adjusted for chronological age, age^2^, sex, and study region, unless the variable was plotted as the x-axis. ProtAgeGap: Difference between proteomic age and chronological age.

# Supplementary Figure 8. Performance and pairwise correlations of overall and organ-specific ageing clocks in the holdout test set.

Note. Correlations between overall and organ-specific ProtAge and ChronAge, as well as pairwise correlations between overall and organ-specific AgeGaps, trained based on Olink proteins only (a & b); SomaScan proteins only (c & d); both Olink and SomaScan proteins (e & f). All analyses were performed in holdout test data (n = 1,193). ProtAgeGap: Difference between ProtAge and ChonAge.

# Supplementary Figure 9. Performance and pairwise correlations of overall and organ-specific ageing clocks in the holdout test set in subcohort participants.

Note. Correlations between overall and organ-specific ProtAge and ChronAge, as well as pairwise correlations between overall and organ-specific AgeGaps, trained based on Olink proteins only (a & b); SomaScan proteins only (c & d); c. both Olink and SomaScan proteins (e & f). All analyses were performed in holdout test data in subcohort participants (n = 608). The thyroid ageing clock trained based on Olink and SomaScan was not available, as Boruta did not select any features. ProtAgeGap: Difference between ProtAge and ChonAge.

# Supplementary Figure 10. Performance and pairwise correlations of overall and organ-specific ageing clocks in the UK Biobank.

Note. Correlations between overall and organ-specific ProtAge and ChronAge, as well as pairwise correlations between overall and organ-specific AgeGaps in the UK Biobank (n = 52,667). The ageing clock was trained based on Olink proteins after Boruta feature selection in the China Kadoorie Biobank. ProtAgeGap: Difference between ProtAge and ChonAge.

# Supplementary Figure 11. Associations of overall and organ-specific ProtAgeGaps with disease endpoints and mortality in subcohort participants.

Note. Associations of overall and organ-specific ProtAgeGaps with a. Ischaemic heart disease; b. stroke; c. diabetes; d. chronic liver disease; e. chronic kidney disease; f. all-cause mortality in subcohort participants. All ageing clocks were trained based on both Olink and SomaScan proteins after Boruta feature selection. The thyroid ageing clock was not available, as Boruta did not select any features. Cox regression models were used and adjusted for age, age2, sex, region, education level, smoking, alcohol drinking, physical activity, body mass index (BMI), prevalent diabetes, prevalent diabetes, and hypertension diagnosis at baseline. Cases of ischaemic heart disease with missing data on development date were excluded from the analysis on ischaemic heart disease. Participants with prevalent diabetes, COPD, chronic liver disease, chronic kidney disease, and cancer at baseline were excluded from the corresponding analyses. HR: Hazard ratio. * indicates significant associations after correction for multiple testing with a false discovery rate = 0.05. ProtAgeGap: Difference between proteomic age and chronological age.

# Supplementary Figure 12. Associations of overall and organ-specific ProtAgeGaps with disease endpoints and mortality using models trained in subcohort participants.

Note. Associations of overall and organ-specific ProtAgeGaps with a. Ischaemic heart disease; b. stroke; c. diabetes; d. chronic liver disease; e. chronic kidney disease; f. all-cause mortality. All ageing clocks were trained based on both Olink and SomaScan proteins after Boruta feature selection in subcohort participants, which were then applied to the overall dataset. The thyroid ageing clock was not available, as Boruta did not select any features. Prentice-weighted (for ischaemic heart disease) and standard (for all other outcomes) Cox regression models were used and adjusted for age, age^2^, sex, region, education level, smoking, alcohol drinking, physical activity, body mass index, prevalent diabetes, and hypertension diagnosis at baseline, where appropriate. Cases of ischaemic heart disease with missing data on development date were excluded from the analysis on Ischaemic heart disease. For analyses of diabetes, chronic liver disease, and chronic kidney disease, participants with the corresponding diseases at baseline were excluded. HR: Hazard ratio. * indicates significant associations after correction for multiple testing with a false discovery rate = 0.05. ProtAgeGap: Difference between proteomic age and chronological age.

# Supplementary Figure 13. Associations of overall and organ-specific accelerated ageotypes with disease endpoints and mortality.

Note. Associations of overall and organ-specific accelerated ageotypes with a. Ischaemic heart disease; b. stroke; c. diabetes; d. chronic liver disease; e. chronic kidney disease; f. all-cause mortality. Accelerated agers were defined as those >1.5 standard deviations above the mean of age-adjusted ProtAgeGap. All ageing clocks were trained based on both Olink and SomaScan proteins after Boruta feature selection. Prentice-weighted (for ischaemic heart disease) and standard (for all other outcomes) Cox regression models were used and adjusted for age, age^2^, sex, region, education level, smoking, alcohol drinking, physical activity, body mass index, prevalent diabetes, and hypertension diagnosis at baseline, where appropriate. Cases of ischaemic heart disease with missing data on development date were excluded from the analysis on ischaemic heart disease. For analyses of diabetes, chronic liver disease, and chronic kidney disease, participants with the corresponding diseases at baseline were excluded. HR: Hazard ratio. * indicates significant associations after correction for multiple testing with a false discovery rate = 0.05. ProtAgeGap: Difference between proteomic age and chronological age.

# Supplementary Figure 14. Associations of overall and organ-specific super ageotypes with disease endpoints and mortality.

Note. Associations of overall and organ-specific super ageotypes with a. Ischaemic heart disease; b. stroke; c. diabetes; d. chronic liver disease; e. chronic kidney disease; f. all-cause mortality. Super agers were defined as those > 1.5 standard deviations below the mean of age-adjusted ProtAgeGap. All ageing clocks were trained based on both Olink and SomaScan proteins after Boruta feature selection. Prentice-weighted (for ischaemic heart disease) and standard (for all other outcomes) Cox regression models were used and adjusted for age, age^2^, sex, region, education level, smoking, alcohol drinking, physical activity, body mass index, prevalent diabetes, and hypertension diagnosis at baseline, where appropriate. Cases of ischaemic heart disease with missing data on development date were excluded from the analysis on Ischaemic heart disease. For analyses of diabetes, chronic liver disease, and chronic kidney disease, participants with the corresponding diseases at baseline were excluded. Some results were omitted for chronic kidney disease, as no events occurred among super agers. HR: Hazard ratio. * indicates significant associations after correction for multiple testing with a false discovery rate = 0.05. ProtAgeGap: Difference between proteomic age and chronological age.

# Supplementary Figure 15. Associations of overall and organ-specific ProtAgeGaps with physiological measurements at baseline in subcohort participants.

Note. Associations of overall and organ-specific ProtAgeGaps with a. BMI; b. waist circumference; c. body fat; d. SBP; e. DBP; f. random glucose in subcohort participants. The thyroid ageing clock was not available, as Boruta did not select any features. All ageing clocks were trained based on both Olink and SomaScan proteins after Boruta feature selection. All models were adjusted for age, age^2^, sex, region, education level, smoking, alcohol drinking, physical activity, BMI, prevalent diabetes, and hypertension diagnosis at baseline, where appropriate. BMI: Body mass index. SBP: systolic blood pressure. DBP: Diastolic blood pressure. * indicates significant associations after correction for multiple testing with a false discovery rate = 0.05. ProtAgeGap: Difference between proteomic age and chronological age.

# Supplementary Figure 16. Associations of overall and organ-specific ProtAgeGaps with physiological measurements at baseline using models trained in subcohort participants.

Note. Associations of overall and organ-specific ProtAgeGaps with a. BMI; b. waist circumference; c. body fat; d. SBP; e. DBP; f. random glucose. All ageing clocks were trained based on both Olink and SomaScan proteins after Boruta feature selection in subcohort participants, which were then applied to the overall dataset. The thyroid ageing clock was not available, as Boruta did not select any features. models were adjusted for age, age^2^, sex, region, education level, smoking, alcohol drinking, physical activity, BMI, prevalent diabetes, and hypertension diagnosis at baseline, where appropriate. BMI: Body mass index. SBP: systolic blood pressure. DBP: Diastolic blood pressure. * indicates significant associations after correction for multiple testing with a false discovery rate = 0.05. ProtAgeGap: Difference between proteomic age and chronological age.

# Supplementary Figure 17. Associations of overall and organ-specific accelerated ageotypes with physiological measurements at baseline.

Note. Associations of overall and organ-specific accelerated ageotypes with a. BMI; b. waist circumference; c. body fat; d. SBP; e. DBP; f. random glucose. Accelerated agers were defined as those > 1.5 standard deviations above the mean of age-adjusted ProtAgeGap. All ageing clocks were trained based on both Olink and SomaScan proteins after Boruta feature selection. All models were adjusted for age, age^2^, sex, region, education level, smoking, alcohol drinking, physical activity, BMI, prevalent diabetes, and hypertension diagnosis at baseline, where appropriate. BMI: Body mass index. SBP: systolic blood pressure. DBP: Diastolic blood pressure. * indicates significant associations after correction for multiple testing with a false discovery rate = 0.05. ProtAgeGap: Difference between proteomic age and chronological age.

# Supplementary Figure 18. Associations of overall and organ-specific super ageotypes with physiological measurements at baseline.

Note. Associations of overall and organ-specific super ageotypes with a. BMI; b. waist circumference; c. body fat; d. SBP; e. DBP; f. random glucose. Super agers were defined as those > 1.5 standard deviations below the mean of age-adjusted ProtAgeGap. All ageing clocks were trained based on both Olink and SomaScan proteins after Boruta feature selection. All models were adjusted for age, age^2^, sex, region, education level, smoking, alcohol drinking, physical activity, BMI, prevalent diabetes, and hypertension diagnosis at baseline, where appropriate. BMI: Body mass index. SBP: systolic blood pressure. DBP: Diastolic blood pressure. * indicates significant associations after correction for multiple testing with a false discovery rate = 0.05. ProtAgeGap: Difference between proteomic age and chronological age.

# Supplementary Figure 19. Associations of overall and organ-specific ProtAgeGaps with ageing-related traits at baseline in subcohort participants.

Note. Associations of overall and organ-specific ProtAgeGaps with a. frailty index (scaled by 10 to aid interpretation); b. slow walking pace; c. self-rated poor health; d. comparative worse health in subcohort participants. All ageing clocks were trained based on both Olink and SomaScan proteins after Boruta feature selection. The thyroid ageing clock was not available, as Boruta did not select any features. All models were adjusted for age, age^2^, sex, region, education level, smoking, alcohol drinking, physical activity, BMI, prevalent diabetes, and hypertension diagnosis at baseline. * indicates significant associations after correction for multiple testing with a false discovery rate = 0.05. ProtAgeGap: Difference between proteomic age and chronological age.

# Supplementary Figure 20. Associations of overall and organ-specific ProtAgeGaps with ageing-related traits at baseline using models trained in subcohort participants.

Note. Associations of overall and organ-specific ProtAgeGaps with a. frailty index (scaled by 10 to aid interpretation); b. slow walking pace; c. self-rated poor health; d. comparative worse health. All ageing clocks were trained based on both Olink and SomaScan proteins after Boruta feature selection in subcohort participants, which were then applied to the overall dataset. The thyroid ageing clock was not available, as Boruta did not select any features. All models were adjusted for age, age^2^, sex, region, education level, smoking, alcohol drinking, physical activity, BMI, prevalent diabetes, and hypertension diagnosis at baseline. * indicates significant associations after correction for multiple testing with a false discovery rate = 0.05. ProtAgeGap: Difference between proteomic age and chronological age. ProtAgeGap: Difference between proteomic age and chronological age.

# Supplementary Figure 21. Associations of overall and organ-specific accelerated ageotypes with ageing-related traits at baseline.

Note. Associations of overall and organ-specific accelerated ageotypes with a. frailty index (scaled by 10 to aid interpretation); b. slow walking pace; c. self-rated poor health; d. comparative worse health. Accelerated agers were defined as those > 1.5 standard deviations above the mean of age-adjusted ProtAgeGap. All ageing clocks were trained based on both Olink and SomaScan proteins after Boruta feature selection. All models were adjusted for age, age^2^, sex, region, education level, smoking, alcohol drinking, physical activity, BMI, prevalent diabetes, and hypertension diagnosis at baseline. * indicates significant associations after correction for multiple testing with a false discovery rate = 0.05. ProtAgeGap: Difference between proteomic age and chronological age. ProtAgeGap: Difference between proteomic age and chronological age.

# Supplementary Figure 22. Associations of overall and organ-specific super ageotypes with ageing-related traits at baseline.

Note. Associations of overall and organ-specific super ageotypes with a. frailty index (scaled by 10 to aid interpretation); b. slow walking pace; c. self-rated poor health; d. comparative worse health. Super agers were defined as those > 1.5 standard deviations below the mean of age-adjusted ProtAgeGap. All ageing clocks were trained based on both Olink and SomaScan proteins after Boruta feature selection. All models were adjusted for age, age^2^, sex, region, education level, smoking, alcohol drinking, physical activity, BMI, prevalent diabetes, and hypertension diagnosis at baseline. * indicates significant associations after correction for multiple testing with a false discovery rate = 0.05. ProtAgeGap: Difference between proteomic age and chronological age. ProtAgeGap: Difference between proteomic age and chronological age.

# Supplementary Figure 23. Associations of overall and organ-specific ProtAgeGaps with resurvey characteristics.

Note. Associations of conventional and organ-specific ProtAgeGaps with a. broadband ultrasound attenuation; b. stiffness index; c. hand grip strength; d. carotid intima-media thickness; e. carotid plaque score; f. bradycardia/heart block (ECG); g. ischaemia (ECG); h. left ventricular hypertrophy (ECG). All models were trained based on both Olink and SomaScan proteins after Boruta feature selection. All models were adjusted for age, age^2^, sex, region, education level, smoking, alcohol drinking, physical activity, BMI, prevalent diabetes, and hypertension diagnosis at baseline. ECG: Electrocardiography. * indicates significant associations after correction for multiple testing with a false discovery rate = 0.05. ProtAgeGap: Difference between proteomic age and chronological age.

# Supplementary Figure 24. Associations of overall and organ-specific ProtAgeGaps with resurvey characteristics in subcohort participants.

Note. Associations of conventional and organ-specific ProtAgeGaps with a. broadband ultrasound attenuation; b. stiffness index; c. hand grip strength; d. carotid intima-media thickness; e. carotid plaque score; f. bradycardia/heart block (ECG); g. ischaemia (ECG); h. left ventricular hypertrophy (ECG) in subcohort participants. All models were trained based on both Olink and SomaScan proteins after Boruta feature selection. The thyroid ageing clock was not available, as Boruta did not select any features. All models were adjusted for age, age^2^, sex, region, education level, smoking, alcohol drinking, physical activity, BMI, prevalent diabetes, and hypertension diagnosis at baseline. ECG: Electrocardiography. * indicates significant associations after correction for multiple testing with a false discovery rate = 0.05. ProtAgeGap: Difference between proteomic age and chronological age.

# Supplementary Figure 25. Associations of overall and organ-specific accelerated ageotypes with resurvey characteristics.

Note. Associations of conventional and organ-specific accelerated ageotypes with a. broadband ultrasound attenuation; b. stiffness index; c. hand grip strength; d. carotid intima-media thickness; e. carotid plaque score; f. bradycardia/heart block (ECG); g. ischaemia (ECG); h. left ventricular hypertrophy (ECG). Accelerated agers were defined as those > 1.5 standard deviations above the mean of age-adjusted ProtAgeGap. All models were trained based on both Olink and SomaScan proteins after Boruta feature selection. All models were adjusted for age, age2, sex, region, education level, smoking, alcohol drinking, physical activity, BMI, prevalent diabetes, and hypertension diagnosis at baseline. ECG: Electrocardiography. * indicates significant associations after correction for multiple testing with a false discovery rate = 0.05. ProtAgeGap: Difference between proteomic age and chronological age.

# Supplementary Figure 26. Associations of overall and organ-specific super ageotypes with resurvey characteristics.

Note. Associations of conventional and organ-specific super ageotypes with a. broadband ultrasound attenuation; b. stiffness index; c. hand grip strength; d. carotid intima-media thickness; e. carotid plaque score; f. bradycardia/heart block (ECG); g. ischaemia (ECG); h. left ventricular hypertrophy (ECG). Super agers were defined as those > 1.5 standard deviations below the mean of age-adjusted ProtAgeGap. All models were trained based on both Olink and SomaScan proteins after Boruta feature selection. All models were adjusted for age, age2, sex, region, education level, smoking, alcohol drinking, physical activity, BMI, prevalent diabetes, and hypertension diagnosis at baseline. Some results were omitted for bradycardia/heart block, as no among super agers with this trait were identified. ECG: Electrocardiography. * indicates significant associations after correction for multiple testing with a false discovery rate = 0.05. ProtAgeGap: Difference between proteomic age and chronological age.

# Supplementary Figure 27. Associations of overall and organ-specific ProtAgeGaps with resurvey characteristics using models trained in subcohort participants.

Note. Associations of conventional and organ-specific ProtAgeGaps with a. broadband ultrasound attenuation; b. stiffness index; c. hand grip strength; d. carotid intima-media thickness; e. carotid plaque score; f. bradycardia/heart block (ECG); g. ischaemia (ECG); h. left ventricular hypertrophy (ECG). All ageing clocks were trained based on both Olink and SomaScan proteins after Boruta feature selection in subcohort participants, which were then applied to the overall dataset. The thyroid ageing clock was not available, as Boruta did not select any features. All models were adjusted for age, age^2^, sex, region, education level, smoking, alcohol drinking, physical activity, BMI, prevalent diabetes, and hypertension diagnosis at baseline. ECG: Electrocardiography. * indicates significant associations after correction for multiple testing with a false discovery rate = 0.05. ProtAgeGap: Difference between proteomic age and chronological age.

# Supplementary Figure 28. Relationships between ProtAgeGaps and age-related diseases and traits.

Note. Associations of overall and organ-specific ProtAgeGaps with a. disease endpoints and mortality, b. physiological measurements at baseline. c. ageing-related traits at baseline. The width of each chord indicates the strength of the association, defined by its -log10(p-value). Only FDR-significant associations are shown here. ProtAgeGap: Difference between proteomic age and chronological age.

# Supplementary Figure 29. Relationships between ProtAgeGaps and resurvey traits.

Note. Associations of overall and organ-specific ProtAgeGaps with resurvey traits. The width of each chord indicates the strength of the association, defined by its -log10(p-value). Only FDR-significant associations are shown here. ProtAgeGap: Difference between proteomic age and chronological age.

# Supplementary Figure 30. Regional plot of the locus at AFF3 associated with overall ProtAgeGap.

Note. Associations with ProtAgeGap are shown ±500kb around the lead variant (rs77668309) that reached the genome-wide significance threshold. The proteomic ageing clock was trained based on both Olink and SomaScan proteins after Boruta feature selection. Genomic positions are shown based on GRCh38. ProtAgeGap: Difference between proteomic age and chronological age.

# Supplementary Figure 31. Regional plot of the locus near IL1RAPL1 associated with overall ProtAgeGap.

Note. Associations with ProtAgeGap are shown ±500kb around the lead variant (rs188694865) that reached the genome-wide significance threshold. The proteomic ageing clock was trained based on both Olink and SomaScan proteins after Boruta feature selection. Genomic positions are shown based on GRCh38. ProtAgeGap: Difference between proteomic age and chronological age.

# Supplementary Figure 32. Manhattan and Q-Q plots of genome-wide associations with adipose ProtAgeGap.

Note. a. Manhattan plot. b. Q-Q plot. Genome-wide association analysis was adjusted for age, age^2^, sex, region, and the first 11 genomic principal components. The dotted line indicates the genome-wide significance threshold (5 × 10^−8^). The proteomic ageing clock was trained based on both Olink and SomaScan proteins after Boruta feature selection. Lead variants were identified with a sliding window of 500 kb and annotated with the genes they are located in or next to. ProtAgeGap: Difference between proteomic age and chronological age.

# Supplementary Figure 33. Manhattan and Q-Q plots of genome-wide associations with adrenal ProtAgeGap.

Note. a. Manhattan plot. b. Q-Q plot. The analysis was adjusted for age, age^2^, sex, region, and the first 11 genomic principal components. The dotted line indicates the genome-wide significance threshold (5 × 10^−8^). The proteomic ageing clock was trained based on both Olink and SomaScan proteins after Boruta feature selection. Lead variants were identified with a sliding window of 500 kb and annotated with the genes they are located in or next to. ProtAgeGap: Difference between proteomic age and chronological age.

# Supplementary Figure 34. Manhattan and Q-Q plots of genome-wide associations with oesophagus ProtAgeGap.

Note. a. Manhattan plot. b. Q-Q plot. The analysis was adjusted for age, age^2^, sex, region, and the first 11 genomic principal components. The dotted line indicates the genome-wide significance threshold (5 × 10^−8^). The proteomic ageing clock was trained based on both Olink and SomaScan proteins after Boruta feature selection. Lead variants were identified with a sliding window of 500 kb and annotated with the genes they are located in or next to. ProtAgeGap: Difference between proteomic age and chronological age.

# Supplementary Figure 35. Manhattan and Q-Q plots of genome-wide associations with immune ProtAgeGap.

Note. a. Manhattan plot. b. Q-Q plot. The analysis was adjusted for age, age^2^, sex, region, and the first 11 genomic principal components. The dotted line indicates the genome-wide significance threshold (5 × 10^−8^). The proteomic ageing clock was trained based on both Olink and SomaScan proteins after Boruta feature selection. Lead variants were identified with a sliding window of 500 kb and annotated with the genes they are located in or next to. ProtAgeGap: Difference between proteomic age and chronological age.

# Supplementary Figure 36. Manhattan and Q-Q plots of genome-wide associations with intestine ProtAgeGap.

Note. a. Manhattan plot. b. Q-Q plot. The analysis was adjusted for age, age^2^, sex, region, and the first 11 genomic principal components. The dotted line indicates the genome-wide significance threshold (5 × 10^−8^). The proteomic ageing clock was trained based on both Olink and SomaScan proteins after Boruta feature selection. Lead variants were identified with a sliding window of 500 kb and annotated with the genes they are located in or next to. ProtAgeGap: Difference between proteomic age and chronological age.

# Supplementary Figure 37. Manhattan and Q-Q plots of genome-wide associations with kidney ProtAgeGap.

Note. a. Manhattan plot. b. Q-Q plot. The analysis was adjusted for age, age^2^, sex, region, and the first 11 genomic principal components. The dotted line indicates the genome-wide significance threshold (5 × 10^−8^). The proteomic ageing clock was trained based on both Olink and SomaScan proteins after Boruta feature selection. Lead variants were identified with a sliding window of 500 kb and annotated with the genes they are located in or next to. ProtAgeGap: Difference between proteomic age and chronological age.

# Supplementary Figure 38. Manhattan and Q-Q plots of genome-wide associations with liver ProtAgeGap.

Note. a. Manhattan plot. b. Q-Q plot. The analysis was adjusted for age, age^2^, sex, region, and the first 11 genomic principal components. The dotted line indicates the genome-wide significance threshold (5 × 10^−8^). The proteomic ageing clock was trained based on both Olink and SomaScan proteins after Boruta feature selection. Lead variants were identified with a sliding window of 500 kb and annotated with the genes they are located in or next to. ProtAgeGap: Difference between proteomic age and chronological age.

# Supplementary Figure 39. Manhattan and Q-Q plots of genome-wide associations with lung ProtAgeGap.

Note. a. Manhattan plot. b. Q-Q plot. The analysis was adjusted for age, age^2^, sex, region, and the first 11 genomic principal components. The dotted line indicates the genome-wide significance threshold (5 × 10^−8^). The proteomic ageing clock was trained based on both Olink and SomaScan proteins after Boruta feature selection. Lead variants were identified with a sliding window of 500 kb and annotated with the genes they are located in or next to. ProtAgeGap: Difference between proteomic age and chronological age.

# Supplementary Figure 40. Manhattan and Q-Q plots of genome-wide associations with pancreas ProtAgeGap.

Note. a. Manhattan plot. b. Q-Q plot. The analysis was adjusted for age, age^2^, sex, region, and the first 11 genomic principal components. The dotted line indicates the genome-wide significance threshold (5 × 10^−8^). The proteomic ageing clock was trained based on both Olink and SomaScan proteins after Boruta feature selection. Lead variants were identified with a sliding window of 500 kb and annotated with the genes they are located in or next to. ProtAgeGap: Difference between proteomic age and chronological age.

# Supplementary Figure 41. Manhattan and Q-Q plots of genome-wide associations with pituitary ProtAgeGap.

Note. a. Manhattan plot. b. Q-Q plot. The analysis was adjusted for age, age^2^, sex, region, and the first 11 genomic principal components. The dotted line indicates the genome-wide significance threshold (5 × 10^−8^). The proteomic ageing clock was trained based on both Olink and SomaScan proteins after Boruta feature selection. Lead variants were identified with a sliding window of 500 kb and annotated with the genes they are located in or next to. ProtAgeGap: Difference between proteomic age and chronological age.

# Supplementary Figure 42. Manhattan and Q-Q plots of genome-wide associations with skin ProtAgeGap.

Note. a. Manhattan plot. b. Q-Q plot. The analysis was adjusted for age, age^2^, sex, region, and the first 11 genomic principal components. The dotted line indicates the genome-wide significance threshold (5 × 10^−8^). The proteomic ageing clock was trained based on both Olink and SomaScan proteins after Boruta feature selection. Lead variants were identified with a sliding window of 500 kb and annotated with the genes they are located in or next to. ProtAgeGap: Difference between proteomic age and chronological age.

# Supplementary Figure 43. Manhattan and Q-Q plots of genome-wide associations with stomach ProtAgeGap.

Note. a. Manhattan plot. b. Q-Q plot. The analysis was adjusted for age, age^2^, sex, region, and the first 11 genomic principal components. The dotted line indicates the genome-wide significance threshold (5 × 10^−8^). The proteomic ageing clock was trained based on both Olink and SomaScan proteins after Boruta feature selection. Lead variants were identified with a sliding window of 500 kb and annotated with the genes they are located in or next to. ProtAgeGap: Difference between proteomic age and chronological age.

# Supplementary Figure 44. Manhattan and Q-Q plots of genome-wide associations with brain ProtAgeGap.

Note. a. Manhattan plot. b. Q-Q plot. The analysis was adjusted for age, age^2^, sex, region, and the first 11 genomic principal components. The dotted line indicates the genome-wide significance threshold (5 × 10^−8^). The proteomic ageing clock was trained based on both Olink and SomaScan proteins after Boruta feature selection. Lead variants were identified with a sliding window of 500 kb and annotated with the genes they are located in or next to. ProtAgeGap: Difference between proteomic age and chronological age.

# Supplementary Figure 45. Associations of overall and organ-specific ProtAgeGaps (Residuals) with disease endpoints and mortality using models trained in all participants and applied to all participants.

Note. Associations of overall and organ-specific ProtAgeGaps with a. Ischaemic heart disease; b. stroke; c. diabetes; d. chronic liver disease; e. chronic kidney disease; f. all-cause mortality. All ageing clocks were trained based on both Olink and SomaScan proteins in all participants, which were then applied to the overall dataset. Prentice-weighted (for ischaemic heart disease) and standard (for all other outcomes) Cox regression models were used and adjusted for age, age^2^, sex, region, education level, smoking, alcohol drinking, physical activity, body mass index, prevalent diabetes, and hypertension diagnosis at baseline, where appropriate. Cases of ischaemic heart disease with missing data on development date were excluded from the analysis on Ischaemic heart disease. For analyses of diabetes, chronic liver disease, and chronic kidney disease, participants with the corresponding diseases at baseline were excluded. HR: Hazard ratio. * indicates significant associations after correction for multiple testing with a false discovery rate = 0.05. ProtAgeGap: Residuals from a regression of proteomic age ~ chronological age.

# Supplementary Figure 46. Associations of overall and organ-specific ProtAgeGaps (Residuals) with physiological measurements at baseline trained in all participants and applied to all participants.

Note. Associations of overall and organ-specific ProtAgeGaps with a. BMI; b. waist circumference; c. body fat; d. SBP; e. DBP; f. random glucose. All ageing clocks were trained based on both Olink and SomaScan proteins in all participants which were then applied to the overall dataset. Models were adjusted for age, age^2^, sex, region, education level, smoking, alcohol drinking, physical activity, BMI, prevalent diabetes, and hypertension diagnosis at baseline, where appropriate. BMI: Body mass index. SBP: systolic blood pressure. DBP: Diastolic blood pressure. * indicates significant associations after correction for multiple testing with a false discovery rate = 0.05. ProtAgeGap: Residuals from a regression of proteomic age ~ chronological age.

# Supplementary Figure 47. Associations of overall and organ-specific ProtAgeGaps (Residuals) with ageing-related traits at baseline using models trained in all participants and applied to all participants.

Note. Associations of overall and organ-specific ProtAgeGaps with a. frailty index (scaled by 10 to aid interpretation); b. slow walking pace; c. self-rated poor health; d. comparative worse health. All ageing clocks were trained based on both Olink and SomaScan proteins in all participants which were then applied to the overall dataset. All models were adjusted for age, age^2^, sex, region, education level, smoking, alcohol drinking, physical activity, BMI, prevalent diabetes, and hypertension diagnosis at baseline. * indicates significant associations after correction for multiple testing with a false discovery rate = 0.05. ProtAgeGap: Residuals from a regression of proteomic age ~ chronological age.

# Supplementary Figure 48. Associations of overall and organ-specific ProtAgeGaps (Residuals) with resurvey characteristics using models trained in all participants and applied to all participants.

Note. Associations of conventional and organ-specific ProtAgeGaps with a. broadband ultrasound attenuation; b. stiffness index; c. hand grip strength; d. carotid intima-media thickness; e. carotid plaque score; f. bradycardia/heart block (ECG); g. ischaemia (ECG); h. left ventricular hypertrophy (ECG). All ageing clocks were trained based on both Olink and SomaScan proteins in all participants which were then applied to the overall dataset. All models were adjusted for age, age^2^, sex, region, education level, smoking, alcohol drinking, physical activity, BMI, prevalent diabetes, and hypertension diagnosis at baseline. ECG: Electrocardiography. * indicates significant associations after correction for multiple testing with a false discovery rate = 0.05. ProtAgeGap: Residuals from a regression of proteomic age ~ chronological age.

# Supplementary Figure 49. Associations of overall and organ-specific ProtAgeGaps (Residuals) with disease endpoints and mortality using models trained in all participants and applied to subcohort participants.

Note. Associations of overall and organ-specific ProtAgeGaps with a. Ischaemic heart disease; b. stroke; c. diabetes; d. chronic liver disease; e. chronic kidney disease; f. all-cause mortality. All ageing clocks were trained based on both Olink and SomaScan proteins in all participants, which were then applied to the subcohort dataset. Prentice-weighted (for ischaemic heart disease) and standard (for all other outcomes) Cox regression models were used and adjusted for age, age^2^, sex, region, education level, smoking, alcohol drinking, physical activity, body mass index, prevalent diabetes, and hypertension diagnosis at baseline, where appropriate. Cases of ischaemic heart disease with missing data on development date were excluded from the analysis on Ischaemic heart disease. For analyses of diabetes, chronic liver disease, and chronic kidney disease, participants with the corresponding diseases at baseline were excluded. HR: Hazard ratio. * indicates significant associations after correction for multiple testing with a false discovery rate = 0.05. ProtAgeGap: Residuals from a regression of proteomic age ~ chronological age.

# Supplementary Figure 50. Associations of overall and organ-specific ProtAgeGaps (Residuals) with physiological measurements at baseline trained in all participants and applied to subcohort participants.

Note. Associations of overall and organ-specific ProtAgeGaps with a. BMI; b. waist circumference; c. body fat; d. SBP; e. DBP; f. random glucose. All ageing clocks were trained based on both Olink and SomaScan proteins in all participants which were then applied to the subcohort dataset. Models were adjusted for age, age^2^, sex, region, education level, smoking, alcohol drinking, physical activity, BMI, prevalent diabetes, and hypertension diagnosis at baseline, where appropriate. BMI: Body mass index. SBP: systolic blood pressure. DBP: Diastolic blood pressure. * indicates significant associations after correction for multiple testing with a false discovery rate = 0.05. ProtAgeGap: Residuals from a regression of proteomic age ~ chronological age.

# Supplementary Figure 51. Associations of overall and organ-specific ProtAgeGaps (Residuals) with ageing-related traits at baseline using models trained in all participants and applied to subcohort participants.

Note. Associations of overall and organ-specific ProtAgeGaps with a. frailty index (scaled by 10 to aid interpretation); b. slow walking pace; c. self-rated poor health; d. comparative worse health. All ageing clocks were trained based on both Olink and SomaScan proteins in all participants which were then applied to the subcohort dataset. All models were adjusted for age, age^2^, sex, region, education level, smoking, alcohol drinking, physical activity, BMI, prevalent diabetes, and hypertension diagnosis at baseline. * indicates significant associations after correction for multiple testing with a false discovery rate = 0.05. ProtAgeGap: Residuals from a regression of proteomic age ~ chronological age.

# Supplementary Figure 52. Associations of overall and organ-specific ProtAgeGaps (Residuals) with resurvey characteristics using models trained in all participants and applied to subcohort participants.

Note. Associations of conventional and organ-specific ProtAgeGaps with a. broadband ultrasound attenuation; b. stiffness index; c. hand grip strength; d. carotid intima-media thickness; e. carotid plaque score; f. bradycardia/heart block (ECG); g. ischaemia (ECG); h. left ventricular hypertrophy (ECG). All ageing clocks were trained based on both Olink and SomaScan proteins in all participants which were then applied to the subcohort dataset. All models were adjusted for age, age^2^, sex, region, education level, smoking, alcohol drinking, physical activity, BMI, prevalent diabetes, and hypertension diagnosis at baseline. ECG: Electrocardiography. * indicates significant associations after correction for multiple testing with a false discovery rate = 0.05. ProtAgeGap: Residuals from a regression of proteomic age ~ chronological age.

# Supplementary Figure 53. Associations of overall and organ-specific ProtAgeGaps (Residuals) with disease endpoints and mortality using models trained in subcohort participants and applied to all participants.

Note. Associations of overall and organ-specific ProtAgeGaps with a. Ischaemic heart disease; b. stroke; c. diabetes; d. chronic liver disease; e. chronic kidney disease; f. all-cause mortality. All ageing clocks were trained based on both Olink and SomaScan proteins in subcohort participants, which were then applied to the overall dataset. Prentice-weighted (for ischaemic heart disease) and standard (for all other outcomes) Cox regression models were used and adjusted for age, age^2^, sex, region, education level, smoking, alcohol drinking, physical activity, body mass index, prevalent diabetes, and hypertension diagnosis at baseline, where appropriate. Cases of ischaemic heart disease with missing data on development date were excluded from the analysis on Ischaemic heart disease. For analyses of diabetes, chronic liver disease, and chronic kidney disease, participants with the corresponding diseases at baseline were excluded. HR: Hazard ratio. * indicates significant associations after correction for multiple testing with a false discovery rate = 0.05. ProtAgeGap: Residuals from a regression of proteomic age ~ chronological age.

# Supplementary Figure 54. Associations of overall and organ-specific ProtAgeGaps (Residuals) with physiological measurements at baseline trained in subcohort participants and applied to all participants.

Note. Associations of overall and organ-specific ProtAgeGaps with a. BMI; b. waist circumference; c. body fat; d. SBP; e. DBP; f. random glucose. All ageing clocks were trained based on both Olink and SomaScan proteins in subcohort participants which were then applied to the overall dataset. Models were adjusted for age, age^2^, sex, region, education level, smoking, alcohol drinking, physical activity, BMI, prevalent diabetes, and hypertension diagnosis at baseline, where appropriate. BMI: Body mass index. SBP: systolic blood pressure. DBP: Diastolic blood pressure. * indicates significant associations after correction for multiple testing with a false discovery rate = 0.05. ProtAgeGap: Residuals from a regression of proteomic age ~ chronological age

# Supplementary Figure 55. Associations of overall and organ-specific ProtAgeGaps (Residuals) with ageing-related traits at baseline using models trained in subcohort participants and applied to all participants.

Note. Associations of overall and organ-specific ProtAgeGaps with a. frailty index (scaled by 10 to aid interpretation); b. slow walking pace; c. self-rated poor health; d. comparative worse health. All ageing clocks were trained based on both Olink and SomaScan proteins in subcohort participants which were then applied to the overall dataset. All models were adjusted for age, age^2^, sex, region, education level, smoking, alcohol drinking, physical activity, BMI, prevalent diabetes, and hypertension diagnosis at baseline. * indicates significant associations after correction for multiple testing with a false discovery rate = 0.05. ProtAgeGap: Residuals from a regression of proteomic age ~ chronological age.

# Supplementary Figure 56. Associations of overall and organ-specific ProtAgeGaps (Residuals) with resurvey characteristics using models trained in subcohort participants and applied to all participants.

Note. Associations of conventional and organ-specific ProtAgeGaps with a. broadband ultrasound attenuation; b. stiffness index; c. hand grip strength; d. carotid intima-media thickness; e. carotid plaque score; f. bradycardia/heart block (ECG); g. ischaemia (ECG); h. left ventricular hypertrophy (ECG). All ageing clocks were trained based on both Olink and SomaScan proteins in subcohort participants which were then applied to the overall dataset. All models were adjusted for age, age^2^, sex, region, education level, smoking, alcohol drinking, physical activity, BMI, prevalent diabetes, and hypertension diagnosis at baseline. ECG: Electrocardiography. * indicates significant associations after correction for multiple testing with a false discovery rate = 0.05. ProtAgeGap: Residuals from a regression of proteomic age ~ chronological age.

# Supplementary Figure 57. Associations of overall and organ-specific ProtAgeGaps (Residuals) with disease endpoints and mortality using models trained in subcohort participants and applied to subcohort participants.

Note. Associations of overall and organ-specific ProtAgeGaps with a. Ischaemic heart disease; b. stroke; c. diabetes; d. chronic liver disease; e. chronic kidney disease; f. all-cause mortality. All ageing clocks were trained based on both Olink and SomaScan proteins in subcohort participants, which were then applied to the subcohort dataset. Prentice-weighted (for ischaemic heart disease) and standard (for all other outcomes) Cox regression models were used and adjusted for age, age^2^, sex, region, education level, smoking, alcohol drinking, physical activity, body mass index, prevalent diabetes, and hypertension diagnosis at baseline, where appropriate. Cases of ischaemic heart disease with missing data on development date were excluded from the analysis on Ischaemic heart disease. For analyses of diabetes, chronic liver disease, and chronic kidney disease, participants with the corresponding diseases at baseline were excluded. HR: Hazard ratio. * indicates significant associations after correction for multiple testing with a false discovery rate = 0.05. ProtAgeGap: Residuals from a regression of proteomic age ~ chronological age.

# Supplementary Figure 58. Associations of overall and organ-specific ProtAgeGaps (Residuals) with physiological measurements at baseline trained in subcohort participants and applied to subcohort participants.

Note. Associations of overall and organ-specific ProtAgeGaps with a. BMI; b. waist circumference; c. body fat; d. SBP; e. DBP; f. random glucose. All ageing clocks were trained based on both Olink and SomaScan proteins in subcohort participants which were then applied to the subcohort dataset. Models were adjusted for age, age^2^, sex, region, education level, smoking, alcohol drinking, physical activity, BMI, prevalent diabetes, and hypertension diagnosis at baseline, where appropriate. BMI: Body mass index. SBP: systolic blood pressure. DBP: Diastolic blood pressure. * indicates significant associations after correction for multiple testing with a false discovery rate = 0.05. ProtAgeGap: Residuals from a regression of proteomic age ~ chronological age

# Supplementary Figure 59. Associations of overall and organ-specific ProtAgeGaps (Residuals) with ageing-related traits at baseline using models trained in subcohort participants and applied to subcohort participants.

Note. Associations of overall and organ-specific ProtAgeGaps with a. frailty index (scaled by 10 to aid interpretation); b. slow walking pace; c. self-rated poor health; d. comparative worse health. All ageing clocks were trained based on both Olink and SomaScan proteins in subcohort participants which were then applied to the subcohort dataset. All models were adjusted for age, age^2^, sex, region, education level, smoking, alcohol drinking, physical activity, BMI, prevalent diabetes, and hypertension diagnosis at baseline. * indicates significant associations after correction for multiple testing with a false discovery rate = 0.05. ProtAgeGap: Residuals from a regression of proteomic age ~ chronological age.

# Supplementary Figure 60. Associations of overall and organ-specific ProtAgeGaps (Residuals) with resurvey characteristics using models trained in subcohort participants and applied to subcohort participants.

Note. Associations of conventional and organ-specific ProtAgeGaps with a. broadband ultrasound attenuation; b. stiffness index; c. hand grip strength; d. carotid intima-media thickness; e. carotid plaque score; f. bradycardia/heart block (ECG); g. ischaemia (ECG); h. left ventricular hypertrophy (ECG). All ageing clocks were trained based on both Olink and SomaScan proteins in subcohort participants which were then applied to the subcohort dataset. All models were adjusted for age, age^2^, sex, region, education level, smoking, alcohol drinking, physical activity, BMI, prevalent diabetes, and hypertension diagnosis at baseline. ECG: Electrocardiography. * indicates significant associations after correction for multiple testing with a false discovery rate = 0.05. ProtAgeGap: Residuals from a regression of proteomic age ~ chronological age.

# Supplementary Table 1. Characteristics of study participants at baseline and resurvey by case-subcohort status.

| **Characteristic^a^** | **IHD cases**  **(n = 1,951)** | **Subcohort**  **(n = 2,025)** | **Total**  **(n = 3,976)** |
| --- | --- | --- | --- |
| **Socio-demographics** |  |  |  |
| Mean age (SD), years | 63.7 (9.3) | 51.3 (10.5) | 57.3 (11.7) |
| Female, % | 45.1 | 62.1 | 53.7 |
| Urban residents, % | 47.0 | 50.6 | 48.8 |
| ≥ 6 years of education, % | 37.7 | 52.1 | 45.1 |
| **Physiological measurements, mean (SD)** |  |  |  |
| BMI, kg/m^2^ | 24.0 (3.8) | 23.8 (3.4) | 23.9 (3.6) |
| Waist circumference, mm | 836.4 (105.9) | 802.6 (99.2) | 819.2 (104.0) |
| Body fat, % | 27.0 (9.3) | 28.6 (8.3) | 27.8 (8.8) |
| SBP, mmHg | 146.3 (24.6) | 130.5 (21.4) | 138.3 (24.3) |
| DBP, mmHg | 80.4 (12.8) | 78.0 (11.1) | 79.2 (12.0) |
| Random glucose, mmol/L | 7.2 (3.9) | 6.0 (2.3) | 6.6 (3.3) |
| **Ageing-related traits** |  |  |  |
| Mean frailty index (SD^)b^ | 1.7 (0.6) | 1.3 (0.6) | 1.5 (0.6) |
| Slow walking pace, % | 6.6 | 2.4 | 4.5 |
| Self-rated poor health, % | 14.6 | 8.3 | 11.4 |
| Comparative worse health, % | 19.1 | 13.9 | 16.4 |
| **Lifestyle habits** |  |  |  |
| Regular smoking, % | 35.2 | 25.3 | 30.1 |
| Regular alcohol drinking, % | 15.0 | 15.2 | 15.1 |
| Mean physical activity (SD), MET-h/day | 13.0 (10.5) | 21.3 (14.5) | 17.3 (13.3) |
| Mean food diversity index (SD) | 10.9 (3.5) | 11.3 (3.3) | 11.1 (3.4) |
| Mean Lifestyle index (SD) | 2.4 (1.0) | 2.8 (1.0) | 2.6 (1.0) |
| **Sleep and mental health** |  |  |  |
| Mean sleep duration (SD), hour | 7.2 (1.5) | 7.5 (1.4) | 7.3 (1.5) |
| Sleep problems, % | 20.6 | 14.9 | 17.7 |
| Depressive symptoms, % | 3.0 | 2.7 | 2.9 |
| Anxiety symptoms, % | 3.0 | 3.3 | 3.1 |
| **Medical history** |  |  |  |
| Prevalent diabetes, % | 16.1 | 6.4 | 11.2 |
| Hypertension diagnosis, % | 25.1 | 9.8 | 17.3 |
| **Resurvey^c^** |  |  |  |
| Broadband ultrasound attenuation, dB/MHz | 106.9 (13.1) | 109.8 (12.5) | 109.0 (12.7) |
| Mean stiffness index (SD) | 83.6 (17.8) | 89.5 (20.6) | 87.8 (20.0) |
| Mean hand grip strength (SD), kg | 23.3 (10.0) | 24.8 (9.3) | 24.4 (9.5) |
| Mean carotid intima‐media thickness (SD), mm | 0.9 (0.2) | 0.8 (0.2) | 0.8 (0.2) |
| Carotid plaque score | 1.6 (1.3) | 0.8 (1.1) | 1.0 (1.2) |
| Bradycardia/heart block (ECG), % | 10.5 | 6.4 | 7.6 |
| Ischaemia (ECG), % | 48.5 | 27.1 | 33.2 |
| Left ventricular hypertrophy (ECG), % | 19.5 | 13.8 | 15.4 |

Note. a. There was 1 case and 3 subcohort participants with missing data on body fat;11 cases and 20 subcohort participants with missing data on random glucose. b. Frailty index was scaled by 10 to aid interpretation. c. Resurvey data were available for 203 cases and 507 subcohort participants, including 4 cases and 7 subcohort participants with missing data on broadband ultrasound attenuation and stiffness index; 7 cases and 9 subcohort participants with missing data on hand grip strength; 2 cases and 4 subcohort participants with missing data on carotid intima‐media thickness; 2 cases and 1 subcohort participant with missing data on carotid plaque score; 3 cases and 6 subcohort participants with missing data on ECG measures. IHD: ischaemic heart disease. BMI: body mass index. SBP: systolic blood pressure. DBP: diastolic blood pressure. ECG: traits defined based on electrocardiography.

# Supplementary Table 2. Performance of Cox risk prediction models for all-cause mortality using conventional risk factors and overall and organ-specific ProtAgeGaps, assessed by C-statistics and Net Reclassification Index (all participants).

| **Model** | **C-statistic (95% CI)** | **100 × Change in C (95% CI)** | **NRI (95% CI)** |
| --- | --- | --- | --- |
| Base model* | 0.876 (0.863, 0.889) | - | - |
| Base model + ProtAgeGap |  |  |  |
| Heart | 0.882 (0.869, 0.895) | 0.6 (0.3, 0.9) | 28 (22, 35) |
| Salivary | 0.880 (0.868, 0.893) | 0.4 (0.2, 0.7) | 25 (19, 32) |
| Liver | 0.880 (0.867, 0.893) | 0.4 (0.2, 0.6) | 26 (19, 33) |
| Pancreas | 0.880 (0.867, 0.892) | 0.4 (0.2, 0.5) | 28 (22, 35) |
| Stomach | 0.879 (0.867, 0.891) | 0.3 (0.1, 0.5) | 20 (15, 26) |
| Immune | 0.879 (0.866, 0.892) | 0.3 (0.1, 0.5) | 25 (19, 32) |
| Brain | 0.878 (0.865, 0.891) | 0.2 (0.0, 0.4) | 26 (20, 33) |
| Kidney | 0.878 (0.865, 0.891) | 0.2 (0.0, 0.4) | 22 (15, 29) |
| Overall | 0.878 (0.865, 0.890) | 0.2 (0.0, 0.3) | 31 (25, 37) |
| Adrenal | 0.878 (0.865, 0.890) | 0.2 (0.0, 0.3) | 22 (16, 28) |
| Adipose | 0.877 (0.864, 0.890) | 0.1 (0.0, 0.3) | 28 (22, 35) |
| Intestine | 0.877 (0.865, 0.890) | 0.1 (0.0, 0.3) | 26 (19, 33) |
| Muscle | 0.877 (0.864, 0.890) | 0.1 (0.0, 0.2) | 13 (7, 19) |
| Skin | 0.877 (0.865, 0.889) | 0.1 (0.0, 0.2) | 26 (21, 32) |
| Artery | 0.876 (0.864, 0.889) | 0.1 (0.0, 0.1) | 32 (26, 39) |
| Lung | 0.876 (0.863, 0.889) | 0.0 (0.0, 0.1) | 2 (-4, 9) |
| Esophagus | 0.876 (0.863, 0.888) | 0.0 (-0.1, 0.1) | 17 (10, 23) |
| Thyroid | 0.876 (0.863, 0.888) | 0.0 (-0.1, 0.0) | 13 (7, 19) |
| Pituitary | 0.875 (0.862, 0.887) | -0.1 (-0.2, 0.0) | -9 (-15, -2) |

*Base model includes: age, sex, smoking, diabetes, SBP and waist circumference

# Supplementary Table 3. Performance of Cox risk prediction models for ischaemic heart disease using conventional risk factors and overall and organ-specific ProtAgeGaps, assessed by C-statistics and Net Reclassification Index (all participants).

| **Model** | **C-statistic (95% CI)** | **100 × Change in C (95% CI)** | **NRI (95% CI)** |
| --- | --- | --- | --- |
| Base model* | 0.838 (0.827, 0.848) | - | - |
| Base model + ProtAgeGap |  |  |  |
| Salivary | 0.842 (0.832, 0.852) | 0.4 (0.2, 0.6) | 18 (12, 25) |
| Heart | 0.840 (0.830, 0.851) | 0.3 (0.0, 0.5) | 17 (11, 23) |
| Immune | 0.840 (0.830, 0.850) | 0.2 (0.1, 0.4) | 20 (14, 26) |
| Liver | 0.839 (0.829, 0.849) | 0.1 (0.0, 0.3) | 20 (13, 26) |
| Stomach | 0.839 (0.829, 0.849) | 0.1 (0.0, 0.2) | 9 (2, 16) |
| Kidney | 0.839 (0.829, 0.849) | 0.1 (0.0, 0.3) | 27 (20, 33) |
| Adipose | 0.839 (0.828, 0.849) | 0.1 (0.0, 0.3) | 29 (23, 35) |
| Brain | 0.838 (0.828, 0.848) | 0.1 (0.0, 0.2) | 19 (14, 23) |
| Adrenal | 0.838 (0.828, 0.848) | 0.1 (-0.1, 0.2) | 17 (11, 24) |
| Skin | 0.838 (0.828, 0.848) | 0.1 (-0.1, 0.2) | 27 (19, 34) |
| Pancreas | 0.838 (0.828, 0.848) | 0.1 (0.0, 0.1) | 12 (6, 19) |
| Muscle | 0.838 (0.828, 0.848) | 0.0 (-0.1, 0.1) | 7 (1, 14) |
| Overall | 0.838 (0.828, 0.848) | 0.0 (0.0, 0.1) | 20 (14, 27) |
| Lung | 0.838 (0.828, 0.848) | 0.0 (0.0, 0.1) | 5 (-2, 12) |
| Intestine | 0.838 (0.827, 0.848) | 0.0 (-0.1, 0.1) | 19 (14, 25) |
| Thyroid | 0.837 (0.827, 0.848) | 0.0 (0.0, 0.0) | -1 (-6, 5) |
| Artery | 0.837 (0.827, 0.848) | 0.0 (-0.1, 0.0) | 25 (18, 32) |
| Esophagus | 0.837 (0.827, 0.848) | 0.0 (0.0, 0.0) | 2 (-6, 10) |
| Pituitary | 0.837 (0.827, 0.847) | -0.1 (-0.2, 0.1) | -8 (-15, -1) |

*Base model includes: age, sex, smoking, diabetes, SBP and waist circumference

# Supplementary Table 4. Performance of Cox risk prediction models for all-cause mortality using conventional risk factors and overall and organ-specific ProtAgeGaps, assessed by C-statistics and Net Reclassification Index (subcohort only).

| **Model** | **C-statistic (95% CI)** | **100 × Change in C (95% CI)** | **NRI (95% CI)** |
| --- | --- | --- | --- |
| Base model* | 0.810 (0.776, 0.844) | - | - |
| Base model + ProtAgeGap |  |  |  |
| Artery | 0.822 (0.791, 0.854) | 1.2 (0.2, 2.2) | 53 (38, 67) |
| Liver | 0.820 (0.787, 0.853) | 1.0 (0.0, 2.0) | 29 (13, 44) |
| Brain | 0.820 (0.787, 0.852) | 1.0 (0.0, 1.9) | 37 (24, 51) |
| Immune | 0.819 (0.784, 0.854) | 0.9 (-0.1, 1.9) | 32 (18, 47) |
| Pancreas | 0.819 (0.788, 0.850) | 0.9 (-0.1, 1.9) | 43 (30, 56) |
| Stomach | 0.818 (0.788, 0.848) | 0.8 (-0.2, 1.8) | 23 (8, 39) |
| Kidney | 0.817 (0.783, 0.851) | 0.7 (-0.2, 1.5) | 28 (12, 44) |
| Adrenal | 0.815 (0.781, 0.849) | 0.5 (-0.1, 1.2) | 29 (13, 45) |
| Salivary | 0.814 (0.779, 0.849) | 0.4 (-0.1, 0.9) | 14 (-3, 31) |
| Overall | 0.814 (0.781, 0.847) | 0.4 (-0.2, 1.0) | 19 (3, 35) |
| Intestine | 0.814 (0.783, 0.845) | 0.4 (-0.2, 1.0) | 32 (20, 44) |
| Skin | 0.813 (0.780, 0.846) | 0.3 (-0.2, 0.8) | 35 (19, 52) |
| Heart | 0.812 (0.780, 0.845) | 0.2 (-0.6, 1.1) | 27 (9, 45) |
| Muscle | 0.812 (0.778, 0.847) | 0.2 (-0.3, 0.8) | 20 (5, 35) |
| Pituitary | 0.811 (0.778, 0.844) | 0.1 (-0.1, 0.4) | 23 (10, 36) |
| Lung | 0.811 (0.777, 0.844) | 0.1 (-0.1, 0.3) | 4 (-13, 21) |
| Adipose | 0.810 (0.778, 0.842) | 0.0 (-0.8, 0.8) | 36 (24, 48) |
| Thyroid | 0.810 (0.776, 0.843) | 0.0 (-0.1, 0.1) | 6 (-9, 21) |
| Esophagus | 0.809 (0.775, 0.843) | -0.1 (-0.3, 0.1) | 16 (0, 33) |

*Base model includes: age, sex, smoking, diabetes, SBP and waist circumference

# Supplementary Table 5. Performance of Cox risk prediction models for ischaemic heart disease using conventional risk factors and overall and organ-specific ProtAgeGaps, assessed by C-statistics and Net Reclassification Index (subcohort only).

| **Model** | **C-statistic (95% CI)** | **100 × Change in C (95% CI)** | **NRI (95% CI)** |
| --- | --- | --- | --- |
| Base model* | 0.748 (0.714, 0.782) | - | - |
| Base model + ProtAgeGap |  |  |  |
| Adipose | 0.760 (0.726, 0.793) | 1.2 (-0.1, 2.4) | 39 (26, 53) |
| Salivary | 0.760 (0.728, 0.791) | 1.2 (0.2, 2.1) | 12 (-1, 25) |
| Immune | 0.757 (0.723, 0.791) | 0.9 (-0.2, 1.9) | 15 (0, 30) |
| Brain | 0.756 (0.722, 0.790) | 0.8 (0.0, 1.6) | 26 (12, 39) |
| Artery | 0.756 (0.724, 0.788) | 0.8 (-0.1, 1.7) | 29 (15, 44) |
| Stomach | 0.754 (0.719, 0.790) | 0.6 (-0.3, 1.6) | 13 (-2, 29) |
| Pancreas | 0.754 (0.721, 0.786) | 0.6 (-0.2, 1.3) | 23 (12, 35) |
| Kidney | 0.752 (0.718, 0.786) | 0.4 (-0.3, 1.2) | 23 (7, 38) |
| Muscle | 0.752 (0.718, 0.786) | 0.4 (-0.4, 1.2) | 15 (0, 30) |
| Liver | 0.751 (0.717, 0.785) | 0.3 (-0.6, 1.2) | 13 (-4, 30) |
| Overall | 0.750 (0.716, 0.783) | 0.2 (-0.5, 0.8) | 20 (3, 37) |
| Adrenal | 0.749 (0.716, 0.783) | 0.2 (-0.2, 0.5) | 10 (-4, 24) |
| Thyroid | 0.748 (0.714, 0.782) | 0.0 (-0.4, 0.4) | 10 (-4, 24) |
| Intestine | 0.748 (0.714, 0.782) | 0.0 (-0.6, 0.6) | 18 (4, 32) |
| Heart | 0.748 (0.713, 0.782) | 0.0 (-0.4, 0.3) | 8 (-6, 22) |
| Skin | 0.747 (0.713, 0.781) | -0.1 (-0.4, 0.3) | 17 (0, 34) |
| Esophagus | 0.747 (0.713, 0.781) | -0.1 (-0.2, 0.0) | -14 (-23, -5) |
| Lung | 0.747 (0.713, 0.781) | -0.1 (-0.3, 0.1) | -7 (-22, 8) |
| Pituitary | 0.746 (0.712, 0.780) | -0.2 (-0.4, 0.0) | -16 (-26, -5) |

*Base model includes: age, sex, smoking, diabetes, SBP and waist circumference

# Supplementary Table 6. Summary of lead variants from GWAS of overall and organ-specific ProtAgeGaps.

| **Organ** | **rsID** | **CHR** | **POS** | **EA** | **NEA** | **EAF** | **BETA** | **SE** | **P** | **INFO** | **Nearest gene** | **In ageing clock?*** |
| --- | --- | --- | --- | --- | --- | --- | --- | --- | --- | --- | --- | --- |
| Overall | rs77668309 | 2 | 100002887 | G | T | 0.96 | 1.40 | 0.25 | 2.80E-08 | 0.78 | AFF3 | No |
| Overall | rs188694865 | 23 | 28348172 | A | G | 0.99 | 3.03 | 0.55 | 3.60E-08 | 0.36 | IL1RAPL1 | No |
| Adipose | rs114992350 | 1 | 160900467 | A | G | 0.95 | -2.62 | 0.29 | 1.00E-19 | 0.98 | ITLN1 | Yes |
| Adrenal | - | 7 | 134455763 | T | TA | 0.78 | -0.85 | 0.12 | 1.60E-12 | 0.90 | AKR1B1 | Yes |
| Oesophagus | rs145500511 | 20 | 23873779 | TCTGAGGAGATAGGAGGAG | T | 0.65 | -2.15 | 0.09 | 4.00E-121 | 0.94 | CST5 | Yes |
| Immune | rs9348859 | 6 | 31282200 | C | T | 0.45 | -0.72 | 0.13 | 1.20E-08 | 1.00 | HLA-C | No |
| Intestine | rs943461 | 6 | 33801632 | C | G | 0.87 | 1.30 | 0.19 | 2.50E-12 | 1.00 | MLN | Yes |
| Kidney | rs4704838 | 5 | 157056670 | A | G | 0.45 | -1.96 | 0.13 | 7.60E-48 | 0.82 | HAVCR1 | Yes |
| Kidney | rs62034975 | 16 | 20381093 | G | C | 0.83 | -0.94 | 0.16 | 1.10E-08 | 0.99 | PDILT | No |
| Liver | rs248160 | 5 | 135957823 | C | T | 0.38 | 1.51 | 0.12 | 3.70E-34 | 0.95 | LECT2 | Yes |
| Lung | rs58337722 | 1 | 27367871 | G | T | 0.86 | -1.01 | 0.16 | 7.30E-10 | 0.96 | MAP3K6 | No |
| Lung | rs3741240 | 11 | 62419070 | G | A | 0.59 | 0.72 | 0.11 | 1.70E-10 | 1.00 | SCGB1A1 | Yes |
| Lung | rs78734309 | 17 | 36058081 | A | G | 0.80 | 2.70 | 0.14 | 1.30E-77 | 0.90 | CCL18 | Yes |
| Pancreas | rs17753263 | 2 | 79071002 | T | C | 0.90 | -1.39 | 0.21 | 4.60E-11 | 0.91 | REG1B | Yes |
| Pancreas | rs2855981 | 7 | 142768623 | A | C | 0.74 | -0.81 | 0.14 | 9.30E-09 | 0.97 | PRSS2 | Yes |
| Pituitary | - | 7 | 98651775 | T | TA | 0.25 | -0.90 | 0.15 | 5.80E-09 | 0.83 | NPTX2 | Yes |
| Skin | rs149582705 | 3 | 124712155 | A | T | 1.00 | 5.39 | 0.96 | 2.30E-08 | 0.89 | KALRN | No |
| Skin | rs573622911 | 16 | 27402357 | CT | C | 0.99 | 4.44 | 0.79 | 2.10E-08 | 0.94 | IL21R | No |
| Skin | rs60376046 | 17 | 34351977 | T | G | 0.86 | 1.07 | 0.19 | 3.00E-08 | 0.84 | CCL13 | No |
| Stomach | rs369627716 | 17 | 64276826 | C | T | 1.00 | 5.61 | 0.99 | 1.60E-08 | 0.55 | TEX2 | No |
| Stomach | rs748372 | 21 | 42374278 | C | G | 0.58 | 0.63 | 0.11 | 7.10E-09 | 0.96 | TMPRSS3 | No |

Note. GWAS was conducted using ProtAgeGaps trained based on both Olink and SomaScan proteins after Boruta-feature selection. Genomic positions are shown based on GRCh38. *Indicates whether the protein coded by the nearest gene was included in the corresponding ageing clock. GWAS: Genome-wide association studies. ProtAgeGap: ProtAgeGap: Difference between proteomic age and chronological age. CHR: Chromosome. POS: Position. EA: Effect allele. NEA: Non-effect allele. EAS: Effect allele frequency. SE: Standard error.

**References**

1.	Walters RG, Millwood IY, Lin K, et al. Genotyping and population characteristics of the China Kadoorie Biobank. *Cell Genomics* 2023; **3**(8): 100361.

2.	Delaneau O, Marchini J, McVean GA, et al. Integrating sequence and array data to create an improved 1000 Genomes Project haplotype reference panel. *Nature Communications* 2014; **5**(1): 3934.
